# Do providers use computerized clinical decision support systems? A systematic review and meta-regression of clinical decision support uptake

**DOI:** 10.1186/s13012-022-01199-3

**Published:** 2022-03-10

**Authors:** Andrew Kouri, Janet Yamada, Jeffrey Lam Shin Cheung, Stijn Van de Velde, Samir Gupta

**Affiliations:** 1 Division of Respirology, Department of Medicine, St. Michael’s Hospital, Unity Health Toronto, 6 PGT, 30 Bond St, Toronto, ON Canada; 2 Daphne Cockwell School of Nursing, Faculty of Community Services, Ryerson University, Toronto, ON Canada; 3 Keenan Research Center, Li Ka Shing Knowledge Institute, St. Michaelʼs Hospital, University of Toronto, Toronto, Ontario Canada; 4 Division for Health Services, Norwegian Institute of Public Health, Oslo, Norway; 5 Department of Medicine, University of Toronto, Toronto, ON Canada

## Abstract

**Background:**

Computerized clinical decision support systems (CDSSs) are a promising knowledge translation tool, but often fail to meaningfully influence the outcomes they target. Low CDSS provider uptake is a potential contributor to this problem but has not been systematically studied. The objective of this systematic review and meta-regression was to determine reported CDSS uptake and identify which CDSS features may influence uptake.

**Methods:**

Medline, Embase, CINAHL, and the Cochrane Database of Controlled Trials were searched from January 2000 to August 2020. Randomized, non-randomized, and quasi-experimental trials reporting CDSS uptake in any patient population or setting were included. The main outcome extracted was CDSS uptake, reported as a raw proportion, and representing the number of times the CDSS was used or accessed over the total number of times it could have been interacted with. We also extracted context, content, system, and implementation features that might influence uptake, for each CDSS. Overall weighted uptake was calculated using random-effects meta-analysis and determinants of uptake were investigated using multivariable meta-regression.

**Results:**

Among 7995 citations screened, 55 studies involving 373,608 patients and 3607 providers met full inclusion criteria. Meta-analysis revealed that overall CDSS uptake was 34.2% (95% CI 23.2 to 47.1%). Uptake was only reported in 12.4% of studies that otherwise met inclusion criteria. Multivariable meta-regression revealed the following factors significantly associated with uptake: (1) formally evaluating the availability and quality of the patient data needed to inform CDSS advice; and (2) identifying and addressing other barriers to the behaviour change targeted by the CDSS.

**Conclusions and relevance:**

System uptake was seldom reported in CDSS trials. When reported, uptake was low. This represents a major and potentially modifiable barrier to overall CDSS effectiveness. We found that features relating to CDSS context and implementation strategy best predicted uptake. Future studies should measure the impact of addressing these features as part of the CDSS implementation strategy. Uptake reporting must also become standard in future studies reporting CDSS intervention effects.

**Registration:**

Pre-registered on PROSPERO, CRD42018092337

**Supplementary Information:**

The online version contains supplementary material available at 10.1186/s13012-022-01199-3.

Contributions to the literature

- Existing systematic reviews of computerized clinical decision support systems have revealed only mild to moderate overall effectiveness of these potentially important knowledge translation tools, but none so far has systematically investigated provider uptake of CDSS.
- This systematic review addresses this evidence gap, finding that uptake is seldom reported in the CDSS literature, and generally low (34%) when it is reported.
- Meta-regression analysis revealed that both context- and implementation-related features best predicted uptake.
- Uptake should be a requirement for CDSS intervention reports moving forward, and for CDSSs to reach their full potential, implementation science principles should be better integrated into intervention design and evaluation.

## Background

Effectively translating evidence into clinical practice remains a fundamental challenge in medicine. Computerized clinical decision support systems (CDSSs) represent one approach to this problem that has been studied extensively over the last 20 years. CDSSs are information technology systems that deliver patient-specific recommendations to clinicians to promote improved care [1]. These systems may be integrated into provider electronic health records, accessed through the Internet, or delivered through mobile devices, and have a range of functions including providing point-of-care clinical prediction rules, highlighting guideline-supported management, optimizing drug ordering and documentation, and discouraging potentially harmful practices [2].

While CDSSs are widely considered to be an indispensable knowledge translation tool across care settings [3, 4], existing systematic reviews have demonstrated only small to moderate improvements in the care processes that they target, and even less promising effects on clinical outcomes [2, 5]. This has led to further research seeking to identify and establish specific features or “active ingredients” of CDSSs that might predict intervention success [6–8]. Prior such studies have defined CDSS “success” as a function of their impact on care process and/or clinical or economic outcomes, but impact will always be contingent on the actual *uptake* (i.e. use) of CDSSs, and suboptimal CDSS uptake has often been cited as an important barrier to success [1, 9]. Various general explanations for poor CDSS uptake have been proposed in previous studies, including time and financial constraints, lack of knowledge or confidence in CDSS technology, workflow disruption, perceptions of loss of autonomy, and low usability [10, 11]. However, no reviews to date have systematically investigated the precise effects of these and other potential determinants on CDSS uptake. This represents a significant knowledge gap, and an opportunity to identify barriers which could be specifically targeted in future CDSS design and/or implementation in order to realize the meaningful improvements in downstream clinical outcomes that have mostly eluded CDSSs to date.

The aim of this systematic review and meta-regression was to address this gap by identifying studies that specifically report uptake of CDSSs and isolating CDSS features that are associated with uptake.

## Methods

We report our findings using the Preferred Reporting Items for Systematic Reviews and Meta-Analyses (PRISMA) guidelines, and our methodology followed the guidance provided in the Cochrane Handbook for Systematic Reviews [12, 13].

### Searches

We searched Ovid MEDLINE, EMBASE, CINAHL, and the Cochrane Database of Controlled Trials, from January 2000 to August 2020 (Additional file 1, henceforth referred to as “Appendix”, page 2-3). We included only studies published since the year 2000, as we were primarily interested in computerized CDSS that leveraged modern information technology, and across a period of advancement for both CDSS technology and implementation science. We manually searched reference lists of included studies. The search strategy was developed by a library scientist and refined after peer review by an external library scientist, using PRESS guidelines [14].

### Study inclusion and exclusion criteria

We included studies evaluating the implementation of CDSS in any health condition, across all care settings and in any patient population. Study designs included randomized controlled trials, non-randomized controlled trials, and controlled quasi-experimental designs. Conference abstracts were included. Use of the CDSS had to be voluntary (i.e. clinicians could choose whether or not to activate the CDSS or were given an option to ignore it if it was automatically activated), and quantitative information about user uptake had to be available in the manuscript or its appendices.

We defined CDSS as a computerized system to support clinical decision-making using patient-specific data. The CDSS could be accessible through providers’ electronic health record system or the Internet or delivered using mobile devices. CDSSs could be directed at physicians, nurses, or other allied health professionals. Eligible CDSSs had to be directly related to patient care, whereby simulation studies or studies focusing only on educating clinicians or trainees were excluded.

Our primary outcome of interest was provider uptake of CDSS. Uptake had to be reported as a raw proportion, representing the number of times the CDSS was used or accessed over the total number of eligible times it could have been interacted with. We focused specifically on the uptake of the CDSS and not on downstream process or clinical outcomes. CDSS uptake could be reported at the following levels: the event level (e.g. the proportion of alerts responded to); the patient level (e.g. proportion of patient visits or unique patients that the system was used for); and/or the clinician level (e.g. proportion of eligible providers who interacted with the system at least once).

### Data extraction and quality assessment

A team of 6 reviewers screened abstracts and titles, and then AK and JY independently screened full-text versions of all studies not excluded in the previous step in duplicate to determine final inclusion. Next, AK, JY, and JLSC extracted data from the included articles and all final data were then independently reviewed by AK, with any remaining disagreements resolved through discussion with co-authors (further details in Additional file 1: Appendix, page 4).

We extracted the following data from included studies: study methodology, clinical setting, participant characteristics, CDSS characteristics, primary outcome reported, study duration, and CDSS uptake (if not directly reported, uptake was calculated based on data presented in the study or its appendices). Studies with more than one CDSS arm were extracted as separate trials. We documented the features of each of these CDSSs that might influence uptake. We built an initial feature list based on the GUIDES checklist. The GUIDES checklist is a tool that guides the design and implementation of CDSSs through a set of recommended criteria derived through systematic literature review, feedback from international CDSS experts, consultation with patients and healthcare stakeholders, and pilot testing [15]. In consultation with the creators of the GUIDES checklist, we created practical definitions for each of the checklist elements to facilitate data extraction into “Yes/No” CDSS feature descriptors. We then supplemented these with any additional features that were found in a previously reported modified Delphi process for identifying CDSS features subjectively found to be important by information technology experts [7]. This resulted in a final list of 52 CDSS features potentially associated with uptake, categorized into CDSS context, content, system, and implementation features, in line with the GUIDES checklist (Table 1). Each feature was graded as Yes/No/Unclear/Not Applicable by reviewers, based on information available within the manuscript itself and/or in studies referenced in the main manuscript which provided further CDSS details. Features that contained more than one element (e.g. “was there a study done and was it positive?”) had to meet both elements to be graded as Yes.

**Table 1:** CDSS uptake features

**CDSS context**	
1. Was there a formal process to identify barriers and enablers to current behaviour prior to the CDSS study? e.g. mapping barriers and enablers to intervention components	
2. Was there a previous study using a CDSS targeting the same primary outcome as the current study, and was that outcome significantly improved by CDSS?	
3. Is there a gap between the desired and the baseline clinical behaviour identified by study authors?	
4. Has the availability and quality of the patient data needed to inform the CDSS been formally evaluated? e.g. chart review, validation of patient-facing electronic questionnaires	
5. Does use of the CDSS enable improvement of the quality of patient data compared to current standard of care? e.g. electronic collection of data, including patient-reported outcomes	
6. Was there a formal pre-study evaluation of user perceptions that assessed informational needs and/or perceived benefit to using CDSS, and if so, was it positive?	
7. Was specific additional hardware (other than what was already present as part of usual care) required and available for the CDSS?	
8. Does the use of CDSS negatively impact the function of existing information systems? e.g. causing new technical issues or slower electronic health record function	
9. Was a formal workflow analysis conducted prior to formalization of the intervention and did it demonstrate intervention feasibility?	
10. If a workflow analysis was performed, did it demonstrate that baseline workflow would allow the introduction of the CDSS?	
**CDSS content**	
11. Are developers from an academic centre and report no significant conflict of interest?	
12. Is the CDSS advice based on disease-specific guidelines?	
13. Does the CDSS present its reasoning and/or cite research evidence to the user at the time of advice?	
14. Does the CDSS present the harms/benefits of provided guidance?	
15. Was the CDSS pilot tested and was the accuracy of information specifically assessed?	
16. Was there a post-study evaluation of users and were their information needs addressed?	
17. Does the CDSS clearly explain/indicate why it was triggered for specific patients/situations?	
18. Was there a pre- or post-study evaluation of users and was CDSS information/advice clear?	
19. Is the CDSS advice available in the location and software system in which it will be implemented?	
20. Does the CDSS advice contradict any current guidelines?	
21. Were there any issues with the amount of decision support delivered if the CDSS was pilot tested?	
**CDSS system**	
22. Was there a formal usability evaluation performed for the CDSS and was it found to be usable?	
23. Was there a pre- or post-study evaluation of users and was workflow facilitation found to be positive?	
24. Can the system be customized to provide improved user functionality?	
25. Is the system always up and running?	
26. Was there a pre- or post-study evaluation of users and was the advice delivery format found to be appropriate?	
27. Was there a pre- or post-study evaluation of users and was the visual display/graphic design of CDSS advice found to be appropriate?	
28. If the CDSS used specific functions for prioritized decision support (i.e. pop-ups), were they pilot tested or assessed in a post-study evaluation?	
29. Does the CDSS provide advice directly to users who will be making the relevant clinical decisions?	
30. Does CDSS facilitate collaboration between healthcare providers?	
31. Does the CDSS provide advice at the moment and point-of-need?	
**CDSS implementation**	
32. Was information about the CDSS available to users (i.e. practical instructions)?	
33. Are dedicated personnel and/or web- or paper-based resources available to CDSS users for technical support (i.e. help desk, tech support)?	
34. Was user training provided for the CDSS?	
35. Were other barriers to the behaviour changes being targeted by the CDSS discussed (i.e. medication costs), and if so were strategies implemented to address those barriers?	
36. Was CDSS implemented in temporal steps?	
37. Was CDSS usage and performance evaluated during the study?	
38. If CDSS usage and performance was monitored during the study, were there strategies in place to fix any identified problems?	
39. Were local users consulted during the intervention planning or implementation?	
40. Was there discussion of an overall strategy (i.e. Knowledge Translation strategy) to guide the CDSS initiative?	
**Additional factors from Roshanov et al.**	
41. Was the CDSS developer involved in authorship of the study?	
42. Was CDSS advice provided automatically in the practitioner’s workflow?	
43. Did the CDSS provide advice for patients (i.e. educational material)?	
44. Did the CDSS require a reason for override of use/recommendations?	
45. Does the CDSS have a critiquing function for actions? (i.e. it activates after orders are entered, suggesting they should be cancelled or revised)	
46. Does the practitioner have to enter data directly into the CDSS?	
47. Does the CDSS provide advice or reminders directly to patients (i.e. independent of the clinician)?	
48. Was the CDSS a commercial product?	
49. Did practitioners receive advice directly through an electronic interface?	
50. Did the CDSS target healthcare practitioners other than physicians?	
51. Was periodic performance feedback provided in addition to patient-specific system advice?	
52. Was there a co-intervention in the CDSS group?	

Given that some features were not routinely reported, we also sought to contact study authors to assist in feature classification. We emailed our completed CDSS feature extraction tables to the corresponding authors of each included study, requesting verification. We sent a reminder email to authors two weeks after the initial email. We adjusted our data extraction table to reflect changes suggested and justified by responding study authors. For studies for which we did not receive an author response, features designated “Unclear” were assumed to be “No” for the purposes of data analysis.

Finally, we extracted details required for risk of bias classification for each study, according to criteria discussed in the Cochrane Handbook for Systematic Reviews of Interventions, modified in line with previous systematic reviews of CDSS [6, 16]. Further details are provided in the Additional file 1.

### Data synthesis and analysis

We used a random-effects meta-analysis model to calculate the average uptake proportion across the included studies, weighted by the inverse of sampling variance [17]. A log-odds (logit) transformation was used to better approximate a normal distribution and facilitate meta-analysis of proportions, as is common in the literature [18–20]. Next, we used a mixed-effects meta-analysis model to perform subgroup analysis, calculating the weighted average uptake proportion for each uptake level (event, patient, and clinician) [21, 22]. We expected substantial heterogeneity given the diversity of interventions, context, and diseases in the included studies.

We then performed univariate meta-regression analyses to explore how CDSS features influenced CDSS uptake across the included studies and may be able to account for the anticipated heterogeneity in CDSS uptake [23]. Using only the covariates from univariable analysis with *p* < 0.25 (see Additional file 1: Appendix page 5-7, Table A1), we fitted a multivariable meta-regression model, and then simplified the resulting model using multimodel inference. Multimodel inference fits and compares all possible model permutations using corrected Akaike information criterion (AIC), and demonstrates the relative importance of each included covariate across all possible models [24, 25]. We sought to determine if the simplified multivariable meta-regression model could reduce the anticipated heterogeneity in uptake proportion, and to identify any CDSS features significantly associated with increased uptake of CDSSs across all possible models.

Finally, given the low level of uptake reporting, we found across the CDSS literature, we performed a post-hoc analysis comparing uptake reporting in screened CDSS studies published prior to versus after the 2011 publication of the CONSORT-eHEALTH extrension [26].

The *I*^2^ statistic was used to report heterogeneity across all models. R Software, version 3.6.2 (R Foundation for Statistical Computing, Vienna) was used to perform all statistical analyses. *Rma* function from the *metaphor* library and *metaprop* function from the *meta* library were used to fit models. The *dmetar* library was used for multimodel inference analysis.

## Results

**Fig. 1:**
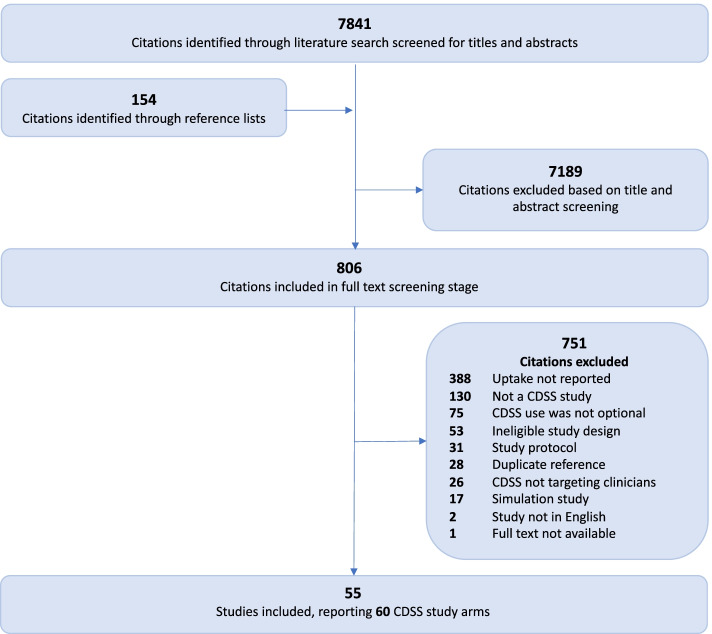
Flow diagram of the review process

Our search identified 7841 unique citations. We identified an additional 154 citations through reference lists of included studies. We excluded 7189 citations at the initial stage of screening, leaving 806 citations (11.2%) for full-text review. Of these, 55 studies (6.8%) met inclusion criteria (see List of Included Trials – Additional file 2). Five included studies had 2 different CDSS arms [27–31], resulting in a total of 60 CDSS study arms (Figure 1). Among 443 studies that met other inclusion criteria, 388 were excluded due to failure to report CDSS uptake, whereby only 55/443 (12.4%) eligible studies reported uptake. Of the 201 eligible studies published in 2011 or earlier (the year of the CONSORT-EHEALTH extension publication), uptake was reported in 24 (11.9%), compared to in 32/242 (13.2%) studies published after 2011 (*p* = 0.77).

Of the 55 included studies, 48 (87.3%) used a randomized design, with cluster randomization being the most common. Most (41/55, 74.6%) were in outpatient settings, were conducted in the USA (36/55, 65.5%), and involved adults (46/55, 83.6%). Uptake was reported at the patient level in 32 (58.2%) studies, at the event level in 16 (29.1%) studies, and at the clinician level in 11 (20.0%) studies (4 studies reported uptake at more than one level) (Table 2). Overall risk of bias was judged to be high in most randomized and non-randomized controlled studies (28/52, 53.9%), unclear in 14/52 studies (26.9%), and low in 10/52 studies (19.2%). Overall risk of bias was low in the 3 interrupted time series studies (Additional file 1: Appendix page 8-11, Fig A1 and Tables A2-A3).

**Table 2:** Summary of characteristics of included studies (*n* = 55)

Study characteristics	Number (%)
Trial design	
Cluster randomized trial	31 (56.4)
Non-cluster randomized trial	17 (30.9)
Non-randomized designs	7 (12.7)
Setting	
Outpatient	41 (74.6)
Inpatient	8 (14,6)
Emergency department	6 (10.9)
Study duration	
Months, mean (SD)	10.3 (5.1)
Location	
USA	36 (65.5)
Other	19 (34.5)
Population	
Adult	46 (83.6)
Paediatric	9 (16.4)
Publication date	
2010 or later	42 (76.4)
Before 2010	13 (23.6)
Uptake type reported^a^	
Patient level	32 (58.2)
Event level	16 (29.1)
Clinician level	11 (20.0)

^a^Some studies reported more than one level of uptake

The 55 included studies covered a broad range of clinical conditions. Most CDSSs were integrated into existing software systems (47/55, 85.5%), were based on disease-specific guidelines (49/55, 89.1%), provided advice at the moment and point of clinical need (52/55, 94.6%), and targeted only physicians (31/55, 56.4%). CDSS developers were the study authors in 48/55 studies (87.3%) (see Full Study Characteristics – Additional file 2).

Authors from 30/55 (54.6%) publications verified our extraction of CDSS features. On average we adjusted answers based on author comments for 8.7 features out of 52, indicating that our baseline assignment of features to “Yes” or “No” was correct 83.3% of the time. Among the 25 studies we did not receive author responses for, we only assigned 1.6/52 (3.1%) features on average as “Unclear”.

The 60 CDSS study arms involved a total of 373,608 patients and 3607 providers. Random effects meta-analysis demonstrated an overall weighted CDSS uptake of 34.2% (95% CI 23.2 to 47.1%). As anticipated, the meta-analysis of uptake proportion across all study arms demonstrated considerable heterogeneity (I^2^ = 99.9%) (Additional file 1: Appendix page 12, Fig A2). Among the different reported uptake types, CDSS uptake at the event level was 26.1% (95% CI 8.2 to 58.3%), at the patient level was 32.9% (95% CI 22.7 to 45.1%), and at the clinician level was 65.6% (95% CI 43.6 to 82.4%). Effect sizes across the different subgroups were significantly different (*Q*(df = 2) = 7.39, *p* = 0.025), but meta-analysis by uptake type did not significantly change the level of heterogeneity (Additional file 1: Appendix page 13, Fig A3). At the request of reviewers, further post-hoc subgroup analyses were conducted to assess uptake based on setting (emergency department, inpatient, or outpatient), provider type (physicians vs. other healthcare providers), disease type (cardiac disease, respiratory disease, infectious disease, or other), and adult versus paediatric patient populations. None of these subgroup analyses resulted in statistically significant differences in effect sizes. A further subgroup meta-analysis was conducted based on risk of bias assessment (high, unclear, low), and this also did not result in statistically significant differences in effect sizes between subgroups (see Additional file 1: Appendix, Page 14 for full results).

We performed outlier influence analysis according to methods detailed by Viechbauer and Cheung [32], which demonstrated that one study contributing two CDSS study arms, Rosenbloom et al., was significantly distorting the pooled uptake estimate. We removed this extreme outlier from our meta-regression analysis, as it would likely have biased results (further details in Additional file 1: Appendix, page 15, text and Fig A4).

Our multivariable meta-regression model controlling for uptake type and other important CDSS features, using only those predictors from univariate screening with *p* < 0.25, and simplified using multimodel inference identified that formally evaluating the availability and quality of the patient data needed to inform CDSS advice (feature 4, *p* = 0.02) and identifying and addressing other barriers to the behaviour change targeted by the CDSS (feature 35, *p* = 0.02) were significantly associated with increased CDSS uptake (Table 3). Heterogeneity remained high with the multivariable meta-regression model (*I*^2^ = 99.5%), but the included covariates accounted for 38.6% of the heterogeneity present in overall uptake proportion (*R*^2^ = 38.6%) (see Additional file 1: Appendix for full univariate analysis results – page 5-7 Table A1, further details regarding model simplification – page 16 text and Fig A5, and uptake feature assignment used in statistical analysis – page 17 Fig A6-A7).

**Table 3:** Multivariable meta-regression model for CDSS uptake, controlling for uptake type (*n* = 58)

CDSS features	Estimate (95% CI)	*p* value
Uptake type		
Clinician	Ref	
Patient	− 1.09 (− 2.63, 0.45)	0.17
Event	− 0.85 (− 3.70, 0.01)	0.34
Feature 4: Has the availability and quality of the patient data needed to inform the CDSS been formally evaluated?		
No (37/58)	Ref	
Yes (21/58)	1.21 (0.24, 2.19)	**0.02**
Feature 35: Were other barriers to the behaviour changes being targeted by the CDSS discussed (i.e. medication costs), and if so were strategies implemented to address those barriers?		
No (51/58)	Ref	
Yes (7/58)	1.64 (0.22, 3.07)	**0.02**
Feature 15: Was the CDSS pilot tested and was the accuracy of information specifically assessed?		
No (40/58)	Ref	
Yes (20/58)	− 0.96 (− 1.98, 0.05)	0.06
Feature 7: Was specific additional hardware (other than what was already present as part of usual care) required and available for the CDSS?		
No (51/58)	Ref	
Yes (7/58)	0.97 (− 0.40, 2.34)	0.17
Feature 44: Did the CDSS require reason for override of use/recommendations?		
No (51/58)	Ref	
Yes (7/58	0.90 (− 0.57, 2.37)	0.23
Feature 52: Was there a co-intervention in the CDSS group?		
No (37/58)	Ref	
Yes (21/58)	0.47 (− 0.53, 1.48)	0.36
Feature 28: If the CDSS used specific functions for prioritized decision support (i.e. pop-ups), were they pilot tested or assessed in a post-study evaluation?		
No (42/58)	Ref	
Yes (15/58)	0.36 (− 0.60, 1.32)	0.46
NA (1/58)		
Feature 38: If CDSS usage and performance was monitored during the study, were there strategies in place to fix any identified problems?		
No (14/58)	Ref	
Yes (19/58)	0.23 (− 0.74, 1.21)	0.64
NA (25/58)		

## Discussion

We performed a systematic review and meta-regression to measure the uptake of CDSSs in published studies, and to identify CDSS features associated with uptake.

The first and most important finding was that CDSS uptake is seldom reported in published studies. Complex interventions such as CDSSs may fail at one of many different levels, and should ideally be accompanied by concurrent process evaluations [33], with the most basic process metric being system uptake [15]. Although uptake is the first and *necessary* step for any behavioural or patient-level outcome changes, only 12.4% of otherwise eligible manuscripts reported uptake. This may reflect a lack of consensus regarding optimal CDSS evaluation and reporting processes. The CONSORT-EHEALTH extension was published in 2011 [26] in an effort to address the unique and complex nature of electronic health (eHealth) interventions such as CDSSs, and does specify a requirement to report uptake of the eHealth intervention being studied. However, uptake reporting was no different in studies published before availability of the CONSORT-EHEALTH extension compared to those published after, and date of publication was not a significant predictor of increased uptake in univariate analysis (Additional file 1: Appendix Table A1). Accordingly, given the importance of system uptake in evaluating reasons for success (and especially failure) of CDSS interventions, our findings suggest an urgent need for broader awareness of CDSS-specific reporting guidelines such as the CONSORT-EHEALTH or newer tools such as the SAFER reporting framework [34], and more strict journal criteria for their use.

Next, our meta-analysis of 60 CDSS study arms involving 373,608 patients and 3607 providers, revealed that overall uptake of CDSS was 34.2%. Considering the significant resources and expenditure involved in implementing a CDSS, the finding that it was used in less than half of potential opportunities is concerning. An expected reporting bias towards more favourable uptake might suggest that true uptake is even lower. Uptake was lowest when reported at the event level (26.1%) and similar at the patient level (32.9%), but higher at the clinician level (65.6%) (*p* = 0.025). This is likely due to the nature of uptake measurement at the clinician level, which represented whether or not clinicians had *ever* used the CDSS during the study period, rather than the reflection of repeated use captured in event- and patient-level measurements. Still, it is compelling that only 2/3rds of clinicians even attempted to use an available CDSS. And among those that did try it, frequency of use clearly remained low. Overall, our findings provide an attractive explanation for the widely reported low effect sizes of CDSSs on process and clinical outcomes [2, 5]. Previous reviews have posited that disappointing CDSS effectiveness might be attributable to the lack of theory-based research, inadequate study duration, or suboptimal intervention-context matching [2, 9, 35]. Ours is the first to systematically assess CDSS uptake rather than more downstream process and clinical outcomes. In doing so, we have identified a broadly important determinant of CDSS effectiveness that suggests a need for greater focus on CDSS implementation design (for initial uptake), in addition to system design (for effective behavioural influence upon sustained usage).

Finally, our multivariable meta-regression identified two CDSS features that significantly predict uptake. The first was a contextual feature: whether a formal evaluation of the availability and quality of patient data needed to inform the CDSS had been performed. The availability and veracity of patient data that is mined from within EHR systems to inform CDSSs could influence uptake both because these data are required for personalized and accurate recommendations to be generated, and because effective access and processing of these data can reduce provider interaction time (by averting the need for provider data entry), which is a recognized barrier to uptake [36]. This finding is particularly relevant as CDSSs are increasingly available as pre-developed “off-the-shelf” interventions, and prospective users will need to carefully examine if these types of CDSSs adequately align with their documentation practices, and patient and data workflows prior to implementation [2]. The second factor, related to CDSS implementation, was the presence of other barriers to the target clinical behaviour and concurrent strategies to address them. This reflects the complexity of real-world clinical behaviours such as test or medication prescribing [37], which are often limited by barriers at system or patient levels, beyond the provider-level memory, time and knowledge barriers typically addressed by CDSSs. It is not suprising that a CDSS would not be used when its target is a behaviour that is prevented by additional barriers that it cannot address. The types of barriers that healthcare providers or organizations should consider when implementing a CDSS include user (both provider and patient) beliefs, attitudes, and skills, professional interactions, clinical capacity and resources, and organizational support [15].

The features identified in our analysis predicting uptake are different from those found to be associated with downstream CDSS effectiveness in previous systematic reviews [6, 7, 38]. This likely reflects the fact that behavioural determinants of choosing to *follow* the recommendations from a CDSS are fundamentally different than those that underpin a clinician’s choice to access and *use* such a system at all. What is most notable about the different features we identified as potential determinants of CDSS uptake is that none were related to the CDSS content or system. These findings are in line with previous qualitative CDSS research that has emphasized the importance of implementation context and attaining good “clinician-patient-system integration” when developing CDSSs [39], and highlight the importance of further integrating implementation science principles into CDSS intervention design [40].

### Risk of bias

Risk of bias in our analysis was concentrated in the minority of trials that were not randomized, and in the risk of contamination category among randomized trials with non-clustered designs (Additional file 1: Appendix, Figure A6 and Table A3). As this type of bias would likely shift the result in favour of increased CDSS uptake, we did not feel that including all trials in our analysis would have changed our overall finding of low CDSS uptake.

### Limitations

The principal limitation of our analysis is the heterogeneity among included trials [2]. Heterogeneity in reported CDSS uptake was considerable, even when analysing the data using uptake subtypes. This was likely a consequence of the diverse nature of CDSS interventions, settings, diseases, and study designs included. We believe that as the first review focused on CDSS uptake, it was important to include a wide breadth of CDSS studies. Although meta-analysis is generally discouraged in this context, we reported the results to provide a weighted summary measure of uptake across the available literature, and to highlight the overall low level of CDSS uptake among studies that report it. We also acknowledge that uptake is but one feature of engagement, which is a multidimensional concept [40]. Furthermore, it is conceivable that tools with relatively low uptake have a behavioural impact through halo reminder and/or education effect on the provider. Secondly, although we developed a broad feature list based on published literature and had a high rate of response from study authors validating our feature assignments, these features accounted for only 40% of the variability in CDSS uptake, suggesting that further study is needed to identify other important elements that drive uptake, which could include the influence of individual conditions or types of outcomes targeted, or more granular details about the system itself, and/or the populations and/or contexts being studied. Also, we acknowledge that like any model not based on a priori hypotheses, our use of multimodel inference to select important CDSS features associated with uptake should be viewed as exploratory and hypothesis generating [25]. We do note that this approach avoids some of the statistical limitations of step-wise variable selection methods [41]. Third, we did not investigate the potential effect of publication bias. However, the large majority of published trials did not report uptake, and unpublished negative studies would be expected to further bias the results towards our finding of low uptake. Finally, our models were limited by the fact that only 12% of studies that otherwise met eligibility criteria reported uptake, and it is possible that other important predictors of CDSS uptake will emerge if and when uptake reporting improves over time. Also, though we strongly believe that efforts to improve uptake of CDSS are clearly required, we acknowledge that future work will be needed to quantitatively establish the anticipated correlation between improved uptake and downstream improvements in process and clinical outcomes, and other study methods such as qualitative inquiry will be necessary to achieve a deeper understanding of how context influences CDSS uptake and implementation.

## Conclusions

Given the often disappointing effects of CDSSs reported across systematic reviews [2, 5], many have called for a re-examination of the role of CDSSs in clinical care [4, 42]. It is possible that future CDSS effectiveness may be improved through technological advancements such as electronic health record interoperability and innovative information technology approaches such as machine learning [4]. However, to realize the benefits of such system-level advancements, existing poor system uptake will first need to be addressed. This will require a dedicated effort to address the determinants of CDSS uptake. Our review is a first step in this process, and identified that contextual and implementation-related factors were most strongly associated with uptake, rather than technological, system, or content features. These findings reinforce the importance of including theory-based implementation science approaches early in CDSS intervention design, in order to realize the promise of ever evolving CDSS technologies. It is also clear that monitoring success in this area will require more consistent reporting of uptake in published CDSS trials.

## Supplementary Information

**Additional file 1.** Supplementary material referenced throughout manuscript.

**Additional file 2.** Full study details. List of included trials and full study characteristics extracted.

## Data Availability

All data generated or analysed during the study are included in the Additional file 1, and any further relevant data are available from the corresponding author on reasonable request.
