# Supplementary material for: Do providers use computerized clinical decision support systems? A systematic review and meta-regression of clinical decision support uptake
Source: Implement Sci. 2022 Mar 10;17:21. doi: 10.1186/s13012-022-01199-3 (PMC8908582; doi:10.1186/s13012-022-01199-3)
Supplement: Supplementary file 1 — Additional file 1. Supplementary material referenced throughout manuscript. [file 13012_2022_1199_MOESM1_ESM.docx]

**Appendix**

**Index:**

Page 2-3: Example search strategies

Page 4: Data extraction, further details

Page 5-7: Full univariate meta-regression results

Page 8-11: Risk of bias full results

Page 12-13: Forest plots for meta-analysis and subgroup meta-analysis

Page 14: Additional Post-Hoc Subgroup Meta-Analyses

Page 15: Outlier influence analysis details

Page 16: Multivariable model selection and simplification using multimodel inference

Page 17: Uptake features assignment for statistical analysis

Page 18: References

**Example search strategies:**

Database: Ovid MEDLINE

1. exp "Appointments and Schedules"/
2. "Forms and Records Control"/
3. Medical Records Systems, Computerized/
4. exp Decision Making, Computer-Assisted/
5. exp Artificial Intelligence/
6. Decision Support Systems, Clinical/
7. Reminder Systems/
8. feedback/
9. Decision Making/ and exp Computers/
10. 1 or 2 or 3 or 4 or 5 or 6 or 7 or 8 or 9
11. (remind$ or sticker$ or (decision adj support) or alert$ or (flowsheet$ or flow sheet$ or flowchart$ or flow chart$) or (order adj1 (form$ or sheet$)) or ((request or encounter) adj1 form$) or checklist$ or ((tag or tagged or sticker or annot$) adj5 (note$ or record$ or sheet$ or chart$ or form$)) or (computer$ adj4 feedback) or (computer$ adj4 suggestion$) or (computer$ adj4 message$) or (computer$ adj4 order) or (electronic adj4 feedback) or (electronic adj4 suggestion$) or (electronic adj4 message$) or (information adj4 feedback) or prompt$).tw.
12. 10 and 11
13. (computer$ adj2 reminder$).tw.
14. 12 or 13
15. randomized controlled trial.pt.
16. controlled clinical trial.pt.
17. randomized controlled trials/
18. random allocation/
19. double blind method/
20. single blind method/
21. clinical trial.pt.
22. exp Clinical Trial/
23. (clinical adj trial?).tw.
24. ((singl$ or doubl$ or trebl$ or tripl$) adj25 (blind$ or mask$)).tw.
25. (random$ or placebo?).tw.
26. 15 or 16 or 17 or 18 or 19 or 20 or 21 or 22 or 23 or 24 or 25
27. animal/
28. human/
29. 27 not (27 and 28)
30. 26 not 29
31. 14 and 30

Result: 2710 references

Database: Ovid EMBASE

1. hospital management/ or exp hospital information system/
2. exp medical record/
3. exp electronic medical record system/
4. exp decision support system/
5. exp artificial intelligence/
6. reminder system/
7. feedback system/
8. exp clinical decision support system/
9. 1 or 2 or 3 or 4 or 5 or 6 or 7 or 8
10. (remind$ or sticker$ or (decision adj support) or alert$ or (flowsheet$ or flow sheet$ or flowchart$ or flow chart$) or (order adj1 (form$ or sheet$)) or ((request or encounter) adj1 form$) or checklist$ or ((tag or tagged or sticker or annot$) adj5 (note$ or record$ or sheet$ or chart$ or form$)) or (computer$ adj4 feedback) or (computer$ adj4 suggestion$) or (computer$ adj4 message$) or (computer$ adj4 order) or (electronic adj4 feedback) or (electronic adj4 suggestion$) or (electronic adj4 message$) or (information adj4 feedback) or prompt$).tw.
11. 9 and 10
12. (computer$ adj2 reminder$).tw.
13. 11 or 12
14. randomized controlled trial/
15. randomization/
16. double blind procedure/
17. single blind procedure/
18. exp clinical trial/
19. (clinical adj trial?).tw.
20. ((singl$ or doubl$ or trebl$ or tripl$) adj25 (blind$ or mask$)).tw.
21. (random$ or placebo?).tw.
22. 14 or 15 or 16 or 17 or 18 or 19 or 20 or 21
23. animal/
24. human/
25. 23 not (23 and 24)
26. 22 not 25
27. 13 and 26

Result: 4964 references

**Data extraction, further details:**

We examined initial search results for duplicates using EndNote^TM^ (Clarivate Analytics, USA). A team of 6 reviewers then met to review screening criteria, and each screened an identical set of 100 randomly selected references (title and abstract only). The test screening exercise resulted in 80% agreement between reviewers and any disagreements were discussed as a team. Reviewers then equally divided and screened the remaining abstracts and titles. AK and JY then independently screened full text versions of all studies not excluded in the previous step in duplicate to determine final inclusion. Any disagreements were resolved through discussion. Next, data extractors (AK, JY, and JLSC) met to co-design a data extraction template (on Excel^TM^) and to discuss CDSS uptake feature definitions. A random sample of 10 of the included studies were then extracted independently by each of the three investigators, with serial reviews and discussion until at least 80% agreement in assignment of uptake features across all reviewers was met. Reviewers then equally divided and extracted data from the remaining included articles. All final data were then independently reviewed by AK, and any remaining disagreements resolved through discussion with co-authors.

**Univariate meta-regression results:**

**Table A1. Complete results of univariate meta-regression analyses for CDSS uptake**

| **Predictor** |  | **Estimate (95% CI)** | **R^2^ (%)** | **P value** |
| --- | --- | --- | --- | --- |
| Trial design | Non-randomized  Randomized | Ref  -0.49 (-1.70, 0.71) | 0.00 | 0.42 |
| Setting | Emergency room  Inpatient  Outpatient | Ref  -0.49 (-2.17, 1.20)  -0.55 (-1.89, 0.79) | 0.00 | 0.57  0.42 |
| Study duration | Months | -0.01 (-0.09, 0.07) | 0.00 | 0.84 |
| Location | Non-US  US | Ref  -0.02 (-0.91, 0.87) | 0.00 | 0.96 |
| Population | Adult  Pediatric | Ref  0.40 (-0.77, 1.48) | 0.00 | 0.53 |
| Publication date | Year | 0.02 (-0.07, 0.10) | 0.00 | 0.71 |
| Uptake type | Clinician  Patient  Event | Ref  -1.44 (-2.77, -0.10)  -1.00 (-2.44, 0.44) | 3.32 | **0.04**  0.17 |
| Feature 1 | No (37/58)  Yes (21/58) | Ref  0.89 (0.06, 1.72) | 7.05 | **0.04** |
| Feature 2 | No (46/58)  Yes (12/58) | Ref  1.41 (0.45, 2.36) | 13.21 | **0.004** |
| Feature 3 | No (3/58)  Yes (55/58) | Ref  0.61 (-1.25, 2.52) | 0.00 | 0.51 |
| Feature 4 | No (37/58)  Yes (21/58) | Ref  0.71 (-0.13, 1.56) | 3.97 | 0.10 |
| Feature 5 | No (26/58)  Yes (32/58) | Ref  0.08 (-0.76, 0.93) | 0.00 | 0.85 |
| Feature 6 | No (42/58)  Yes (16/58) | Ref  1.09 (0.21, 1.97) | 9.85 | **0.02** |
| Feature 7 | No (51/58)  Yes (7/58) | Ref  1.36 (0.13, 2.59) | 6.89 | **0.03** |
| Feature 8 | No (58/58)  Yes (0/58) | NA | NA | NA |
| Feature 9 | No (51/58)  Yes (7/58) | Ref  0.70 (-0.57, 1.96) | 0.59 | 0.28 |
| Feature 10 | No (1/58)  Yes (6/58)  NA (51/60) | Ref  1.91 (-2.10, 5.93) | 0.00 | 0.35 |
| Feature 11 | No (2/58)  Yes (56/58) | Ref  0.48 (-1.80, 2.77) | 0.00 | 0.68 |
| Feature 12 | No (6/58)  Yes (52/58) | Ref  1.11 (-0.23, 2.45) | 2.70 | 0.10 |
| Feature 13 | No (19/58)  Yes (39/58) | Ref  -0.04 (-0.93, 0.85) | 0.00 | 0.93 |
| Feature 14 | No (46/58)  Yes (12/58) | Ref  0.50 (-0.53, 1.54) | 0.00 | 0.34 |
| Feature 15 | No (40/58)  Yes (20/58) | Ref  0.51 (-0.36, 1.38) | 1.27 | 0.25 |
| Feature 16 | No (47/58)  Yes (11/58) | Ref  0.56 (-0.49, 1.61) | 0.56 | 0.30 |
| Feature 17 | No (15/58)  Yes (43/58) | Ref  -0.07 (-1.02, 0.89) | 0.00 | 0.89 |
| Feature 18 | No (42/58)  Yes (16/58) | Ref  -0.002 (-0.94, 0.93) | 0.00 | 0.99 |
| Feature 19 | No (8/58)  Yes (50/58) | Ref  -0.59 (-1.79, 0.61) | 0.10 | 0.34 |
| Feature 20 | No (58/58)  Yes (0/58) | NA | NA | NA |
| Feature 21 | No (53/58)  Yes (5/58) | Ref  -0.19 (-1.68, 1.30) | 0.00 | 0.80 |
| Feature 22 | No (48/58)  Yes (10/58) | Ref  -0.17 (-1.28, 0.93) | 0.00 | 0.76 |
| Feature 23 | No (47/58)  Yes (9/58) | Ref  -0.36 (-1.51, 0.79) | 0.00 | 0.54 |
| Feature 24 | No (54/58)  Yes (4/58) | Ref  0.07 (-1.58, 1.71) | 0.00 | 0.94 |
| Feature 25 | No (58/58)  Yes (0/58) | NA | NA | NA |
| Feature 26 | No (42/58)  Yes (16/58) | Ref  -0.09 (-1.02, 0.85) | 0.00 | 0.86 |
| Feature 27 | No (46/58)  Yes (12/58) | Ref  -0.45 (-1.46, 0.58) | 0.00 | 0.39 |
| Feature 28 | No (42/58)  Yes (15/58)  NA (1/58) | Ref  0.91 (-0.02, 1.84) | 5.61 | 0.06 |
| Feature 29 | No (1/58)  Yes (57/58) | Ref  0.39 (-2.81, 3.59) | 0.00 | 0.81 |
| Feature 30 | No (43/58)  Yes (15/58) | Ref  0.87 (-0.07, 1.81) | 3.76 | 0.07 |
| Feature 31 | No (3/58)  Yes (55/58) | Ref  0.37 (-1.52, 2.25) | 0.00 | 0.70 |
| Feature 32 | No (32/58)  Yes (26/58) | Ref  -0.46 (-1.30, 0.38) | 0.00 | 0.28 |
| Feature 33 | No (46/58)  Yes (22/58) | Ref  0.63 (-0.22, 1.48) | 1.82 | 0.15 |
| Feature 34 | No (16/58)  Yes (42/58) | Ref  0.35 (-0.58, 1.29) | 0.00 | 0.46 |
| Feature 35 | No (51/58)  Yes (7/58) | Ref  0.98 (-0.30, 2.26) | 1.40 | 0.13 |
| Feature 36 | No (49/58)  Yes (9/58) | Ref  0.11 (-1.05, 1.26) | 0.00 | 0.86 |
| Feature 37 | No (25/58)  Yes (33/58) | Ref  0.50 (-0.34, 1.33) | 0.78 | 0.24 |
| Feature 38 | No (14/58)  Yes (19/58)  NA (25/58) | Ref  0.70 (-0.29, 1.69) | 1.83 | 0.17 |
| Feature 39 | No (22/58)  Yes (36/58) | Ref  0.62 (-0.23, 1.47) | 2.24 | 0.15 |
| Feature 40 | No (35/58)  Yes (23/58) | Ref  0.38 (-0.47, 1.23) | 0.00 | 0.38 |
| Feature 41 | No (6/58)  Yes (52/58) | Ref  -0.40 (-1.81, 1.02) | 0.00 | 0.58 |
| Feature 42 | No (12/58)  Yes (46/58) | Ref  0.49 (-0.54, 1.52) | 0.00 | 0.35 |
| Feature 43 | No (42/58)  Yes (16/58) | Ref  0.78 (-0.14, 1.70) | 2.66 | 0.10 |
| Feature 44 | No (51/58)  Yes (7/58) | Ref  1.16 (-0.16, 2.48) | 1.19 | 0.09 |
| Feature 45 | No (47/58)  Yes (11/58) | Ref  -0.61 (-1.67, 0.45) | 1.17 | 0.26 |
| Feature 46 | No (29/58)  Yes (29/58) | Ref  0.06 (-0.78, 0.90) | 0.00 | 0.89 |
| Feature 47 | No (53/58)  Yes (5/58) | Ref  0.50 (-0.98, 1.98) | 0.00 | 0.51 |
| Feature 48 | No (57/58)  Yes (1/58) | Ref  -1.45 (-4.63, 1.74) | 0.00 | 0.37 |
| Feature 49 | No (4/58)  Yes (54/58) | Ref  -0.64 (-2.27, 0.10) | 0.00 | 0.45 |
| Feature 50 | No (34/58)  Yes (24/58) | Ref  0.35 (-0.49, 1.20) | 0.00 | 0.41 |
| Feature 51 | No (40/58)  Yes (18/58) | Ref  0.22 (-0.69, 1.13) | 0.00 | 0.63 |
| Feature 52 | No (37/58)  Yes (21/58) | Ref  0.70 (-0.16, 1.57) | 1.58 | 0.11 |

**Risk of bias:**

We extracted details required for risk of bias classification for each study, according to criteria discussed in the Cochrane Handbook for Systematic Reviews of Interventions, modified in line with previous systematic reviews of CDSS.^2,3^ Specifically, the question of performance bias (i.e. blinding of participants and personnel) was omitted, as blinding is not possible with interventions such as CDSS. After each include risk of bias category was assessed as either high risk, unclear risk, or low risk, a summary assessment was made for each study. If at least one category was rated as high risk, the overall risk was judged to be high. Studies with 2 or less unclear risk gradings and no high risk ones were judged to be low risk overall, and all other were rated as unclear risk. Summary results and individual gradings are presented below in Figure A6 and Table A3 for randomized and non-randomized controlled studies.

**Figure A1. Summary of risk of bias across all randomized and non-randomized controlled trials (n=52)**

**Table A2. Risk of bias assessment for randomized and non-randomized controlled studies (n=52)**

| **+ = low risk**  **- = high risk**  **? = unclear risk** | **Random sequence generation** | **Allocation concealment** | **Similar outcome prevalence at baseline** | **Similar characteristics at baseline** | **Incomplete outcome data** | **Blinding of outcome assessment** | **Adequate protection against contamination** | **Selective reporting** | **Other bias** | **Overall Bias** |
| --- | --- | --- | --- | --- | --- | --- | --- | --- | --- | --- |
| **Anchala 2015** | **?** | **?** | **?** | **+** | **+** | **+** | **+** | **+** | **+** | Unclear |
| **Andruchow 2018** | **?** | **-** | **-** | **+** | **+** | **+** | **?** | **?** | **+** | High |
| **Arts 2017** | **+** | **?** | **?** | **?** | **+** | **+** | **+** | **+** | **+** | Unclear |
| **Atlas 2011** | **+** | **+** | **+** | **-** | **+** | **?** | **+** | **+** | **+** | High |
| **Atlas 2014** | **?** | **?** | **+** | **+** | **+** | **?** | **-** | **?** | **+** | Unclear |
| **Ballard 2016** | **-** | **-** | **+** | **-** | **+** | **+** | **+** | **+** | **+** | High |
| **Blecker 2019** | **+** | **?** | **+** | **+** | **+** | **?** | **-** | **+** | **+** | High |
| **Bosworth 2009** | **?** | **+** | **-** | **+** | **+** | **+** | **-** | **+** | **+** | High |
| **Bourgeois 2010** | **?** | **?** | **?** | **+** | **?** | **+** | **+** | **+** | **+** | Unclear |
| **Co 2010** | **+** | **?** | **+** | **+** | **+** | **?** | **+** | **+** | **+** | Low |
| **Cox 2020** | **+** | **?** | **?** | **+** | **+** | **+** | **+** | **+** | **+** | Low |
| **Diaz 2018** | **?** | **?** | **?** | **?** | **+** | **+** | **-** | **?** | **-** | High |
| **Eckman 2016** | **?** | **?** | **+** | **+** | **?** | **+** | **+** | **+** | **+** | Unclear |
| **Forrest 2013** | **?** | **?** | **-** | **+** | **+** | **+** | **+** | **-** | **+** | High |
| **Goergen 2006** | **-** | **-** | **+** | **+** | **+** | **+** | **?** | **?** | **+** | High |
| **Gonzales 2013** | **?** | **?** | **-** | **-** | **+** | **+** | **-** | **+** | **+** | High |
| **Hendrix 2015** | **+** | **?** | **?** | **?** | **+** | **+** | **+** | **+** | **+** | Unclear |
| **Hetlevik 2000** | **?** | **?** | **+** | **+** | **+** | **?** | **+** | **?** | **-** | High |
| **Hetlevik 1999** | **?** | **?** | **+** | **+** | **+** | **+** | **+** | **?** | **+** | Unclear |
| **Kahan 2017** | **-** | **-** | **?** | **+** | **+** | **?** | **+** | **+** | **+** | High |
| **Kharbanda 2018** | **+** | **?** | **+** | **+** | **+** | **+** | **+** | **+** | **+** | Low |
| **Kuilboer 2006** | **+** | **?** | **+** | **+** | **+** | **+** | **+** | **+** | **+** | Low |
| **Lee 2009** | **?** | **?** | **+** | **+** | **?** | **?** | **-** | **?** | **+** | High |
| **Lester 2006** | **+** | **+** | **+** | **+** | **+** | **?** | **?** | **+** | **+** | Low |
| **Linder 2009** | **?** | **?** | **?** | **+** | **+** | **+** | **+** | **+** | **+** | Unclear |
| **Linder 2009** | **+** | **?** | **+** | **+** | **+** | **+** | **+** | **+** | **+** | Low |
| **Mazzaglia 2016** | **+** | **?** | **+** | **+** | **?** | **?** | **?** | **?** | **+** | Unclear |
| **McDonald 2016** | **+** | **?** | **+** | **?** | **+** | **+** | **+** | **?** | **+** | Unclear |
| **McGinn 2013** | **+** | **?** | **?** | **+** | **+** | **+** | **-** | **+** | **+** | High |
| **McKie 2020** | **+** | **?** | **?** | **?** | **?** | **+** | **+** | **+** | **+** | Unclear |
| **McLaughlin 2010** | **?** | **+** | **?** | **+** | **-** | **?** | **+** | **?** | **+** | High |
| **Meigs 2003** | **+** | **?** | **+** | **+** | **?** | **+** | **?** | **?** | **+** | Unclear |
| **O'Connor 2011** | **?** | **?** | **-** | **?** | **+** | **+** | **+** | **+** | **+** | High |
| **Paulsen 2020** | **+** | **+** | **+** | **+** | **+** | **+** | **-** | **+** | **+** | High |
| **Reed 2018** | **-** | **-** | **?** | **-** | **?** | **+** | **+** | **?** | **+** | High |
| **Reynolds 2020** | **+** | **?** | **?** | **+** | **?** | **?** | **-** | **+** | **+** | High |
| **Rindal 2012** | **?** | **?** | **?** | **+** | **-** | **?** | **+** | **+** | **+** | High |
| **Robbins 2012** | **+** | **+** | **?** | **+** | **-** | **+** | **-** | **+** | **+** | High |
| **Rosenbloom 2005** | **+** | **?** | **?** | **?** | **+** | **-** | **-** | **+** | **+** | High |
| **Samore 2005** | **?** | **?** | **+** | **+** | **+** | **+** | **+** | **+** | **+** | Low |
| **Schnipper 2010** | **+** | **?** | **?** | **+** | **+** | **+** | **-** | **+** | **+** | High |
| **Schwarz 2012** | **?** | **?** | **+** | **?** | **+** | **?** | **-** | **?** | **?** | High |
| **Semler 2015** | **+** | **-** | **+** | **+** | **+** | **+** | **-** | **?** | **+** | High |
| **Silbernagel 2016** | **+** | **?** | **?** | **+** | **+** | **+** | **-** | **+** | **+** | High |
| **Snooks 2014** | **+** | **+** | **?** | **+** | **?** | **+** | **?** | **+** | **+** | Unclear |
| **Spirk 2017** | **+** | **?** | **?** | **+** | **?** | **?** | **-** | **+** | **+** | High |
| **Stockwell 2015** | **?** | **?** | **?** | **+** | **+** | **+** | **-** | **+** | **+** | High |
| **Tamblyn 2015** | **+** | **?** | **?** | **+** | **+** | **+** | **?** | **+** | **+** | Unclear |
| **Tang 2012** | **+** | **?** | **?** | **+** | **+** | **?** | **-** | **+** | **+** | High |
| **Van Wijk 2001** | **+** | **+** | **?** | **+** | **?** | **+** | **+** | **+** | **+** | Low |
| **Williams 2010** | **+** | **+** | **?** | **+** | **+** | **+** | **+** | **+** | **+** | Low |
| **Wright 2012** | **?** | **+** | **+** | **+** | **+** | **+** | **+** | **+** | **+** | Low |

**Table A3. Risk of Bias Assessment in ITS studies using ROBINS-I (n=3)**

| **Risk options:**  Low  Moderate  High  Critical | **Bias due to confounding** | **Bias in selection of participants into the study** | **Bias in classification of interventions** | **Bias due to deviations from intended interventions** | **Bias due to missing data** | **Bias in measurement of the outcome** | **Bias in selection of the reported result** |
| --- | --- | --- | --- | --- | --- | --- | --- |
| **Boutis 2013** | Low | Low | Low | Low | Low | Mod | Low |
| **Gupta 2019** | Low | Low | Low | Low | Mod | Mod | Low |
| **Sheibani 2018** | Low | Low | Low | Low | Mod | Low | Low |


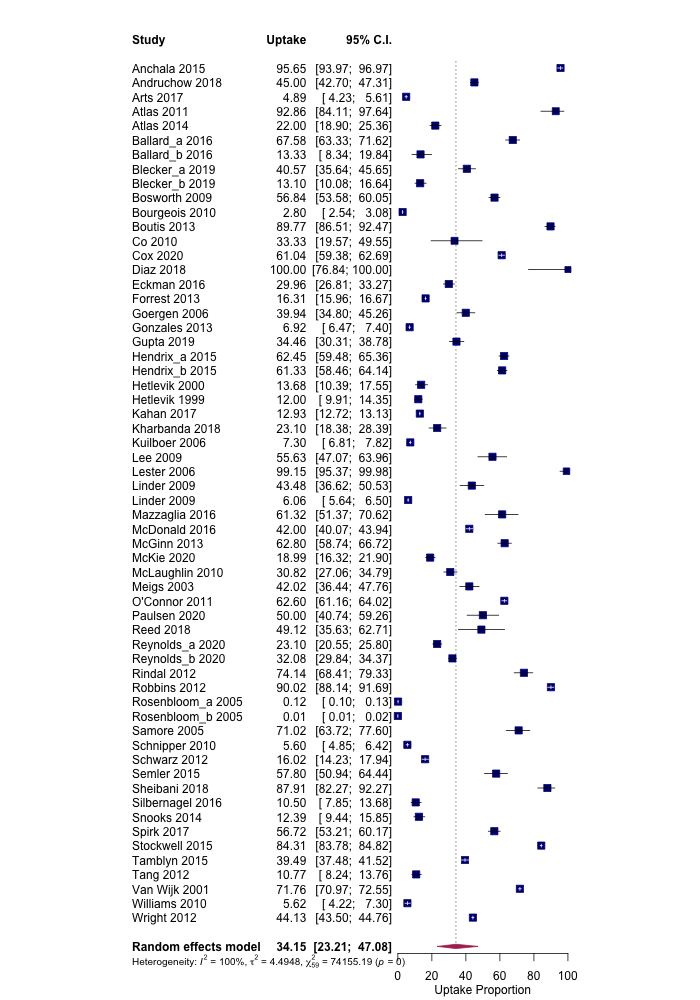


**Figure A2. Random-effects meta-analysis of uptake proportion across all included studies**


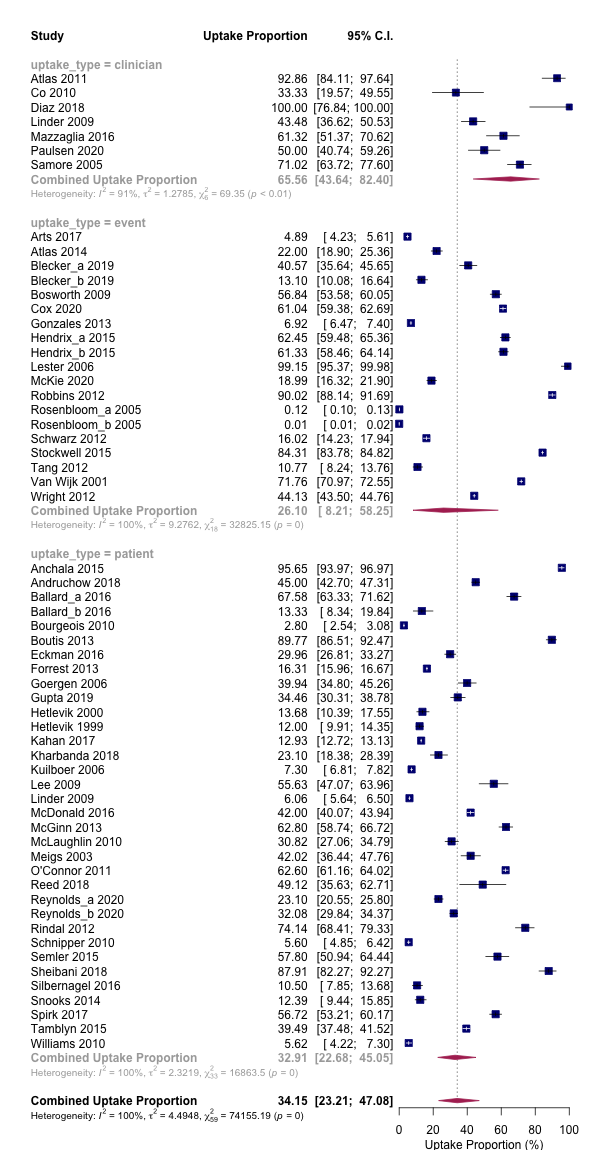


**Figure A3. Subgroup meta-analysis of uptake proportion based on reported uptake type**

**Additional Post-Hoc Subgroup Meta-Analyses:**

The results of reviewer requested post-hoc subgroup meta-analyses did not result in statistically significant between group differences in uptake. Results are presented below:

- CDSS setting (emergency department, inpatient, outpatient): Q(df=2)=2.56, p=0.278
- Practitioner type (physician only vs. other healthcare practitioners): Q(df=1)=2.02, p=0.156
- Disease type (cardiac disease, respiratory disease, infectious disease, other): Q(df=3)=2.69, p=0.442
- Pediatric vs. adult patient population: Q(df=1)=0.98, p=0.322
- Study bias (high, unclear, low): Q(df=2)=0.94, p=0.624

**Outlier Influence Analysis:**

In order to investigate for outlier studies in our meta-analysis, we applied methods proposed by Viechbauer and Cheung.^1^ We first screened the included studies for studentized residuals larger than 3, which identified both trials from Rosenbloom et al. as potential outliers (z-scores were -3.1 and -4.7). We then performed leave-one-out analysis, where each study is removed in turn and the meta-analysis is re-performed, demonstrating the influence of each individual study on the overall effect summary. Once again, both Rosembloom et al. trials had the largest influence on the overall effect summary when removed, shifting the overall uptake up from 34.2% to 36.4% and 37.2%. Further leave-one-out diagnostics detailed in Viechbauer and Cheung were consistent with these findings (see Figure A3 below).


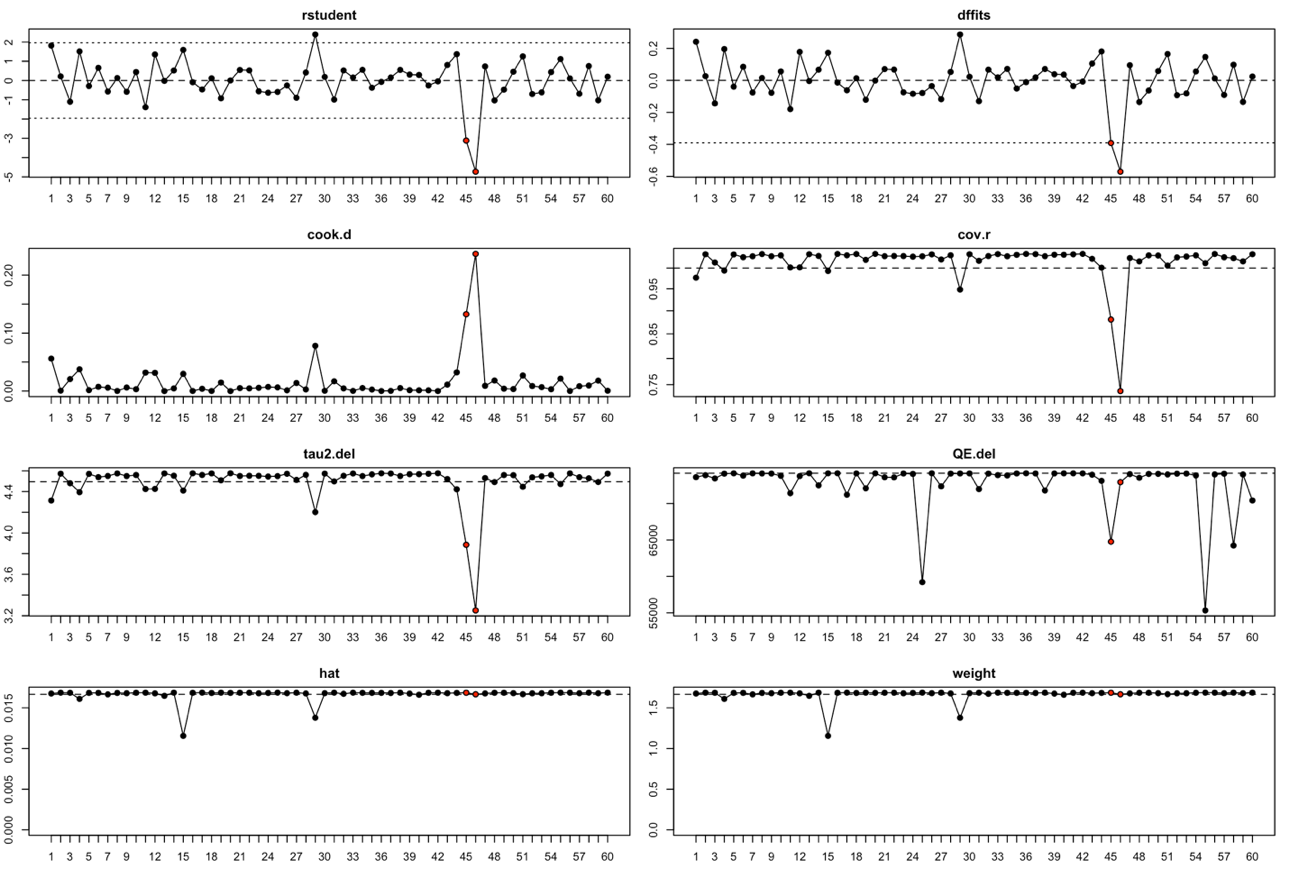


**Figure A4. Full leave-one-out analysis demonstrating that both Rosenbloom et al. trials are significant outliers (represented by the red dots in each graph)**

Reviewing Rosenbloom et al., the CDSS intervention was implemented in 1999 and consisted of nine separate and unrelated decision support features that could be triggered multiple times per electronic order session and per patient. Features were triggered at a mean rate of 1261 times per day, resulting in 418,739 “opportunities” counted for decision support over the study period.^29^ This method or reporting event-level uptake was unique and resulted in a denominator 2 or more orders of magnitude larger than the majority of included studies. Removing this study from the meta-analysis increased the overall CDSS uptake result to 39.6% (95% CI 30.2% to 49.8%). Given its disproportionate influence on pooled uptake estimate relating to the unique method in which uptake was calculated, and the older age of the study, we did not include this extreme outlier in our meta-regression analysis, as it would likely have significantly biased results.

**Multivariable model covariate selection process using multimodel inference:**

Multimodel inference analysis was performed using only those variables with p<0.25 (uptake denominator type, feature 1, feature 2, feature 4, feature 6, feature 7, feature 12, feature 15, feature 28, feature 30, feature 33, feature 35, feature 37, feature 38, feature 39, feature 43, feature 44, feature 52). All possible regression models with these variables were fitted, using the Knapp-Hartung adjustment for computing test statistics, and corrected AIC to compare models. This resulted in 262,144 unique models evaluated. The relative importance of each predictor, reflecting the summed Alaike weights of each predictor across all possible models including that predictor (relative to other predictors), is demonstrated in Figure A4. For the final multivariable model, we included only those predictors that had relative importance above 0.50, and uptake type.


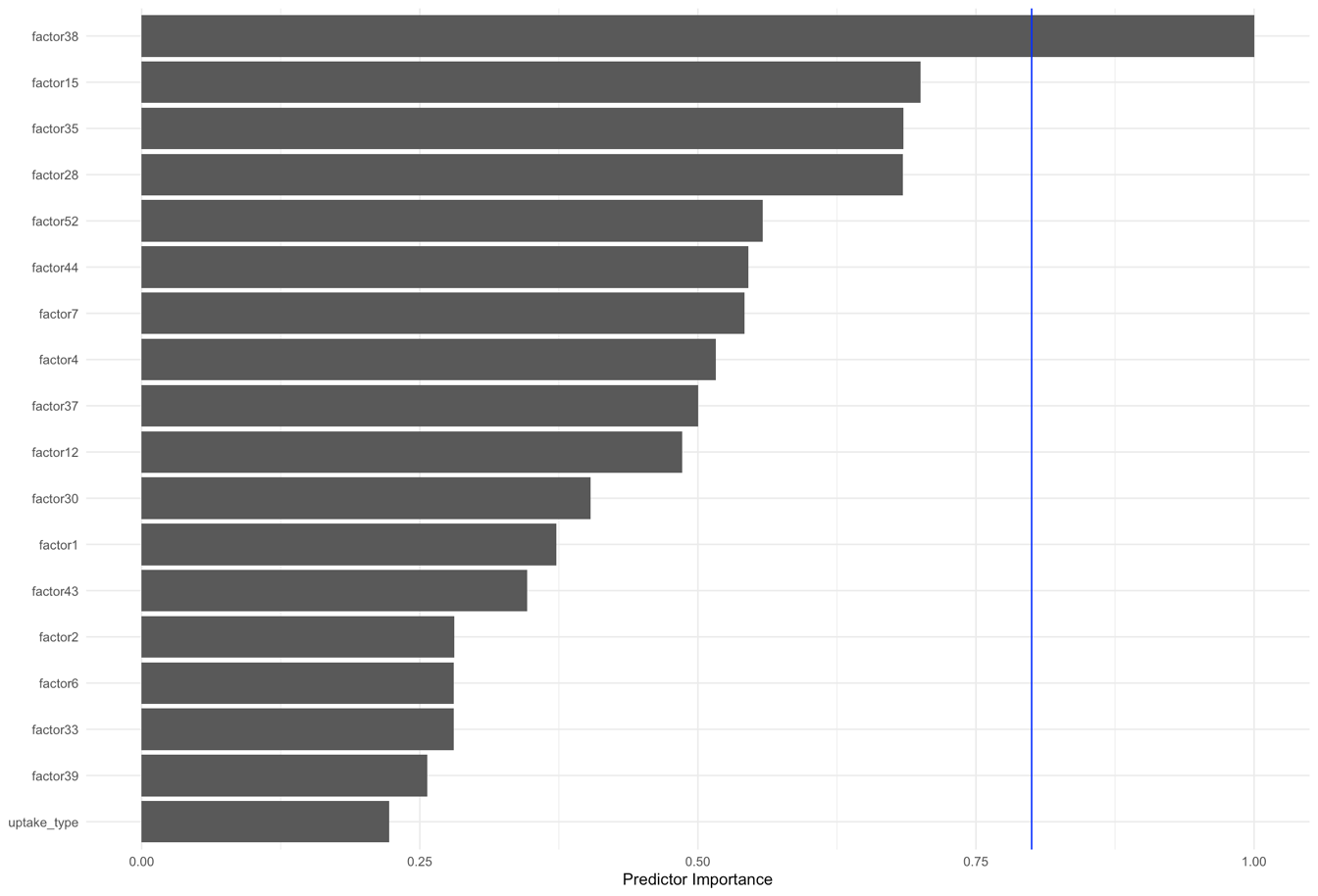


**Figure A5. Relative predictor importance over all possible regression models**

**Uptake features assignments used for statistical analysis:**

**Figure A6. Uptake feature assignment, features 1-30 (1=yes, 0=no)**

**
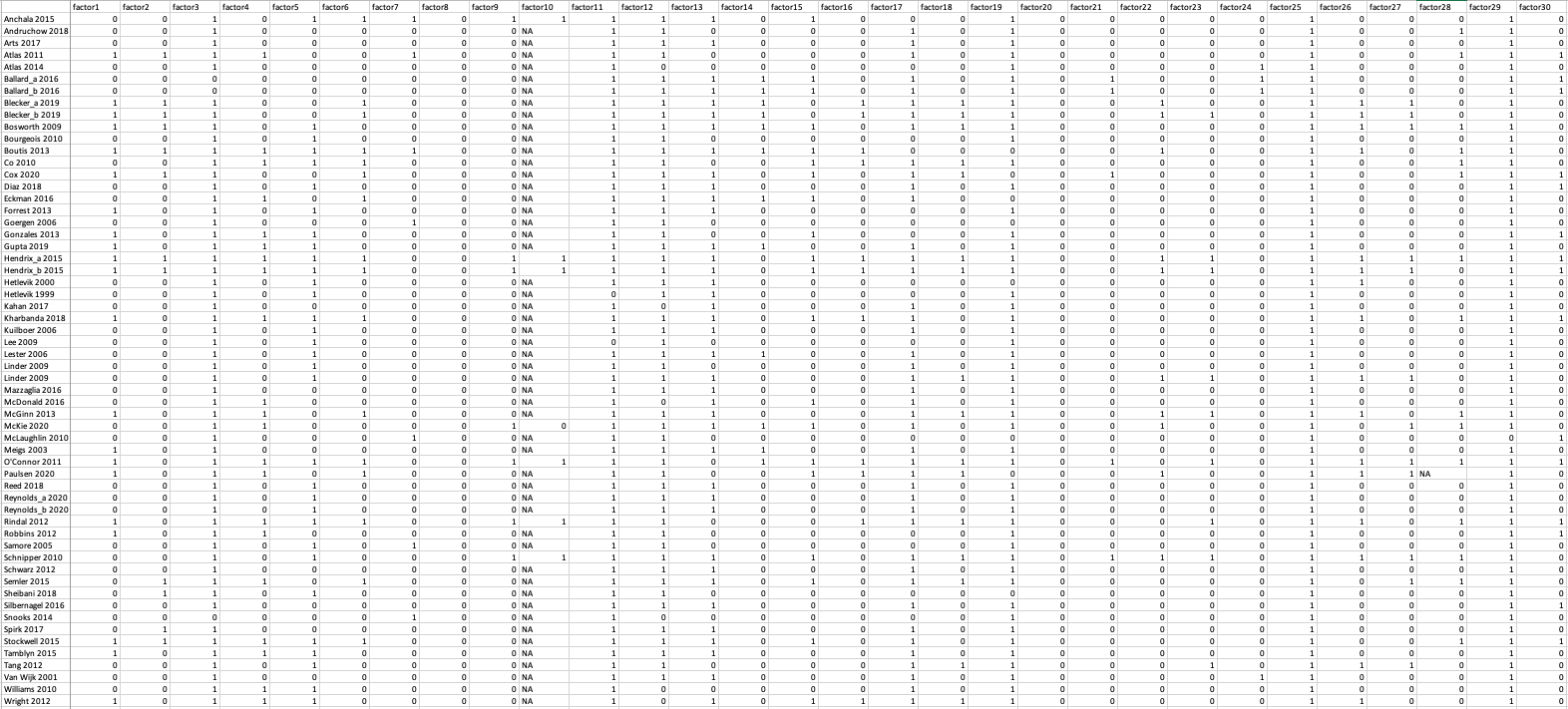
**

**Figure A7. Uptake feature assignment, features 31-52 (1=yes, 0=no)**

**
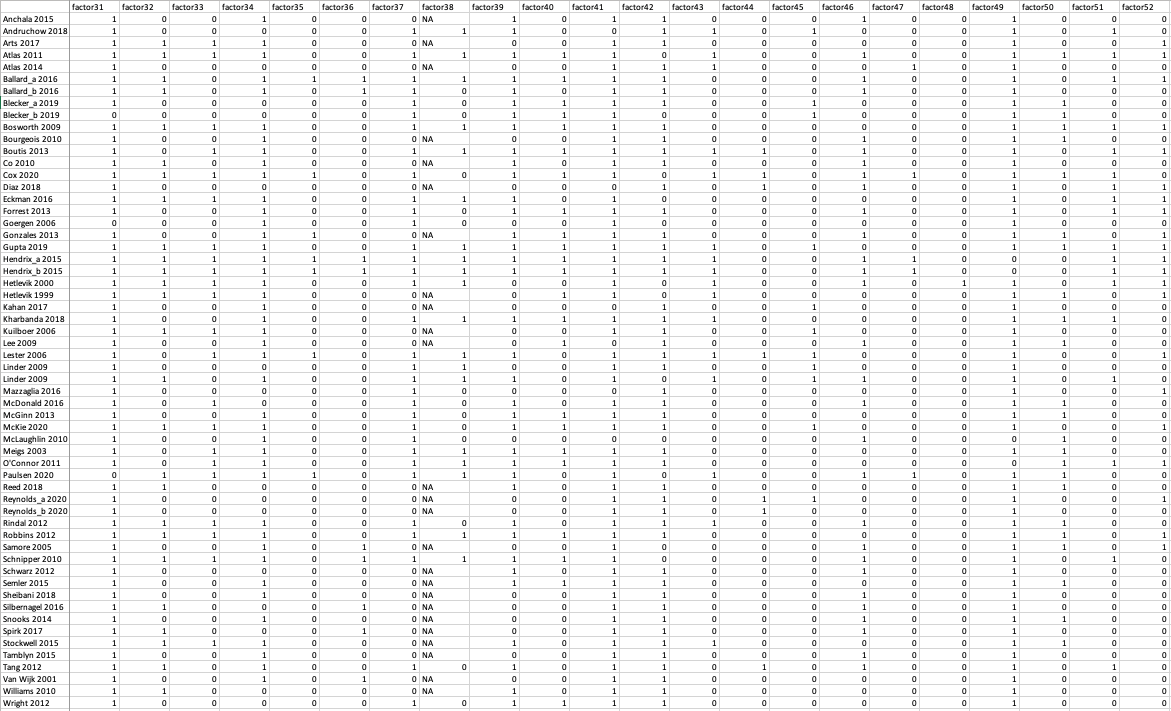
**

**References:**

1. Viechtbauer W, Cheung MW-L. Outlier and influence diagnostics for meta-analysis. Res Synth Methods. 2010;1(2):112–25.

2. Van de Velde S, Heselmans A, Delvaux N, Brandt L, Marco-Ruiz L, Spitaels D, et al. A systematic review of trials evaluating success factors of interventions with computerised clinical decision support. Implement Sci IS [Internet]. 2018 Aug 20 [cited 2019 Nov 13];13. Available from: https://www.ncbi.nlm.nih.gov/pmc/articles/PMC6102833/

3. Higgins JPT, Altman DG, Gøtzsche PC, Jüni P, Moher D, Oxman AD, et al. The Cochrane Collaboration’s tool for assessing risk of bias in randomised trials. BMJ. 2011 Oct 18;343:d5928.
