# Supplementary material for: Do providers use computerized clinical decision support systems? A systematic review and meta-regression of clinical decision support uptake
Source: Implement Sci. 2022 Mar 10;17:21. doi: 10.1186/s13012-022-01199-3 (PMC8908582; doi:10.1186/s13012-022-01199-3)
Supplement: Supplementary file 2 — Additional file 2. Full study details. List of included trials and full study characteristics extracted. [file 13012_2022_1199_MOESM2_ESM.docx]

**Full Study Details**

**List of Included Trials:**

1. Anchala R, et al. Evaluation of effectiveness and cost-effectiveness of a clinical decision support system in managing hypertension in resource constrained primary health care settings: results from a cluster randomized trial. J Am Heart Assoc. 2015;4(1):e001213
2. Andruchow JE, et al. A randomized controlled trial of electronic clinical decision support to reduce unnecessary CT imaging for patients with suspected pulmonary embolism. Canadian Journal of Emergency Medicine. 2018 May;20(S1):S32-3
3. Arts DL, et al. Effectiveness and usage of a decision support system to improve stroke prevention in general practice: a cluster randomized controlled trial. PLoS One. 2017 Feb 28;12(2):e0170974
4. Atlas SJ, et al. A cluster-randomized trial of a primary care informatics-based system for breast cancer screening. Journal of general internal medicine. 2011 Feb;26(2):154-61
5. Atlas, SJ. et al. The medication metronome: A health it system to improve medication management and laboratory monitoring for chronic diseases. In Journal of General Internal Medicine. 2014; Vol. 29, pp. S230-S230
6. Ballard DW, et al. Optimizing clinical decision support in the electronic health record: clinical characteristics associated with the use of a decision tool for disposition of ED patients with pulmonary embolism. Applied clinical informatics. 2016;7(3):883
7. Blecker S, et al. Interrupting providers with clinical decision support to improve care for heart failure. International journal of medical informatics. 2019 Nov 1;131:103956
8. Bosworth HB, et al. Patient education and provider decision support to control blood pressure in primary care: a cluster randomized trial. American heart journal. 2009 Mar 1;157(3):450-6
9. Bourgeois FC, et al. Impact of a computerized template on antibiotic prescribing for acute respiratory infections in children and adolescents. Clinical pediatrics. 2010 Oct;49(10):976-83
10. Boutis K, et al. Effect of the Low Risk Ankle Rule on the frequency of radiography in children with ankle injuries. CMAJ. 2013 Oct 15;185(15):E731-8
11. Co JP, et al. Electronic health record decision support and quality of care for children with ADHD. Pediatrics. 2010 Aug 1;126(2):239-46
12. Cox JL, et a. Integrated Management Program Advancing Community Treatment of Atrial Fibrillation (IMPACT-AF): a cluster randomized trial of a computerized clinical decision support tool. American heart journal. 2020 Jun 1;224:35-46
13. Diaz MC, et al. A Provider-Focused Intervention to Promote Optimal Care of Pediatric Patients With Suspected Elbow Fracture. Pediatric emergency care. 2018 Jan 23
14. Eckman MH, et al. Impact of an atrial fibrillation decision support tool on thromboprophylaxis for atrial fibrillation. American heart journal. 2016 Jun 1;176:17-27
15. Forrest CB, et al. Improving adherence to otitis media guidelines with clinical decision support and physician feedback. Pediatrics. 2013 Apr 1;131(4):e1071-81
16. Goergen SK, et al. Can an evidence‐based guideline reduce unnecessary imaging of road trauma patients with cervical spine injury in the emergency department?. Australasian radiology. 2006 Dec;50(6):563-9
17. Gonzales R, et al. A cluster randomized trial of decision support strategies for reducing antibiotic use in acute bronchitis. JAMA internal medicine. 2013 Feb 25;173(4):267-73
18. Gupta S, et al. The Electronic Asthma Management System (eAMS) improves primary care asthma management. European Respiratory Journal. 2019 Apr 1;53(4)
19. Hendrix KS, et al. Pediatricians' responses to printed clinical reminders: does highlighting prompts improve responsiveness?. Academic pediatrics. 2015 Mar 1;15(2):158-64
20. Hetlevik I, et al. Implementing clinical guidelines in the treatment of diabetes mellitus in general practice: Evaluation of effort, process, and patient outcome related to implementation of a computer-based decision support system. International journal of technology assessment in health care. 2000 Jan;16(1):210-27
21. Hetlevik I, et al. Implementing clinical guidelines in the treatment of hypertension in general practice: evaluation of patient outcome related to implementation of a computer-based clinical decision support system. Scandinavian journal of primary health care. 1999 Jan 1;17(1):35-40
22. Kahan NR, et al. Large-scale, community-based trial of a personalized drug-related problem rectification system. American Journal of Pharmacy. 2017 Mar 1;9(2):41-6
23. Kharbanda EO, et al. Evaluation of an electronic clinical decision support tool for incident elevated BP in adolescents. Academic pediatrics. 2018 Jan 1;18(1):43-50
24. Kuilboer MM, et al. Computed critiquing integrated into daily clinical practice affects physicians’ behavior. Methods of information in medicine. 2006;45(04):447-54
25. Lee NJ, et al. The effect of a mobile clinical decision support system on the diagnosis of obesity and overweight in acute and primary care encounters. Advances in Nursing Science. 2009 Jul 1;32(3):211-21
26. Lester WT, et al. Randomized controlled trial of an informatics-based intervention to increase statin prescription for secondary prevention of coronary disease. Journal of general internal medicine. 2006 Jan;21(1):22-9
27. Linder JA, et al. An electronic health record–based intervention to improve tobacco treatment in primary care: a cluster-randomized controlled trial. Archives of internal medicine. 2009 Apr 27;169(8):781-7
28. Linder JA, et al. Documentation-based clinical decision support to improve antibiotic prescribing for acute respiratory infections in primary care: a cluster randomised controlled trial. Journal of Innovation in Health Informatics. 2009;17(4):231-40
29. Mazzaglia G, et al. Effects of a computerized decision support system in improving pharmacological management in high-risk cardiovascular patients: A cluster-randomized open-label controlled trial. Health Inform J. 2016;22(2):232-47
30. McDonald MV, et al. Outcomes of clinical decision support (CDSS) and correlates of CDSS use for home care patients with high medication regimen complexity: a randomized trial. J Eval Clin Pract. 2016;22(1):10-9
31. McGinn TG, et al. Efficacy of an evidence-based clinical decision support in primary care practices: a randomized clinical trial. JAMA Intern Med. 2013;173(17):1584-91
32. McKie PM, et al. Computerized Advisory decision support for cardiovascular diseases in primary care: a cluster randomized trial. The American journal of medicine. 2020 Jun 1;133(6):750-6
33. McLaughlin D, et al. Office-based interventions for recognizing abnormal pediatric blood pressures. Clinical pediatrics. 2010 Apr;49(4):355-62
34. Meigs JB, et al. A controlled trial of web-based diabetes disease management: the MGH diabetes primary care improvement project. Diabetes Care. 2003 Mar 1;26(3):750-7
35. O'Connor PJ, et al. Impact of electronic health record clinical decision support on diabetes care: a randomized trial. Ann Fam Med. 2011;9(1):12-21
36. Paulsen MM, et al. Effects of using the MyFood decision support system on hospitalized patients' nutritional status and treatment: A randomized controlled trial. Clinical Nutrition. 2020 Dec 1;39(12):3607-17
37. Reed H, et al. Impact of a Best Practice Alert Linking Clostridium difficile Infection Test Results to a Severity-Based Treatment Order Set. In: Open Forum Infectious Diseases 2018 Nov (Vol. 5, No. Suppl 1, p. S79)
38. Reynolds EL, et al. Randomized controlled trial of a clinical decision support system for painful polyneuropathy. Muscle & nerve. 2020 May;61(5):640-4
39. Rindal DB, et al. Computer-assisted guidance for dental office tobacco-cessation counseling: a randomized controlled trial. Am J Prev Med. 2013;44(3):260-4
40. Robbins GK, et al. Efficacy of a clinical decision-support system in an HIV practice: a randomized trial. Annals of Internal Medicine. 2012;157(11):757-66
41. Rosenbloom, ST, et al. 2005 Effect of CPOE user interface design on user-initiated access to educational and patient information during clinical care. Journal of the American Medical Informatics Association. 2005 Jul 1;12(4):458-73
42. Samore MH, et al. Clinical decision support and appropriateness of antimicrobial prescribing: a randomized trial. Jama. 2005 Nov 9;294(18):2305-14
43. Schnipper JL, et al. Effects of documentation-based decision support on chronic disease management. Am J Manag Care. 2010;16(12 Suppl HIT):SP72-81
44. Schwarz EB, et al. Clinical decision support to promote safe prescribing to women of reproductive age: a cluster-randomized trial. Journal of general internal medicine. 2012 Jul 1;27(7):831-8
45. Semler MW, et al. An electronic tool for the evaluation and treatment of sepsis in the ICU: a randomized controlled trial. Critical care medicine. 2015 Aug;43(8):1595
46. Sheibani R, et al. The Effect of a Clinical Decision Support System on Improving Adherence to Guideline in the Treatment of Atrial Fibrillation: An Interrupted Time Series Study. ournal of medical systems. 2018 Feb 1;42(2):26
47. Silbernagel G, et al. Electronic Alert System for Improving Stroke Prevention Among Hospitalized Oral-Anticoagulation-Naïve Patients With Atrial Fibrillation: a Randomized Trial. Journal of the American Heart Association. 2016 Jul 22;5(7):e003776
48. Snooks HA, et al. Support and Assessment for Fall Emergency Referrals (SAFER 1): cluster randomised trial of computerised clinical decision support for paramedics. PloS one. 2014 Sep 12;9(9):e106436
49. Spirk D, et al. Electronic alert system for improving appropriate thromboprophylaxis in hospitalized medical patients: a randomized controlled trial. Journal of thrombosis and haemostasis. 2017 Nov;15(11):2138-46
50. Stockwell MS, et al. Registry-linked electronic influenza vaccine provider reminders: a cluster-crossover trial. Pediatrics. 2015;135(1):e75-82
51. Tamblyn R, et al. Evaluating the impact of an integrated computer-based decision support with person-centered analytics for the management of asthma in primary care: a randomized controlled trial. J Am Med Inform Assoc. 2015;22(4):773-83
52. Tang JW, et al. Electronic tools to assist with identification and counseling for overweight patients: a randomized controlled trial. Journal of general internal medicine. 2012 Aug 1;27(8):933-9
53. Van Wijk MA, et al. Assessment of decision support for blood test ordering in primary care: a randomized trial. Annals of internal medicine. 2001 Feb 20;134(4):274-81
54. Williams LK, et al. A cluster-randomized trial to provide clinicians inhaled corticosteroid adherence information for their patients with asthma. J Allergy Clin Immunol. 2010;126(2):225-31, 31.e1-4
55. Wright A, et al. Improving completeness of electronic problem lists through clinical decision support: a randomized, controlled trial. J Am Med Inform Assoc. 2012;19(4):555-61

**Full Study Characteristics:**

| Anchala R, et al. Evaluation of effectiveness and cost-effectiveness of a clinical decision support system in managing hypertension in resource constrained primary health care settings: results from a cluster randomized trial. J Am Heart Assoc. 2015;4(1):e001213. | |
| --- | --- |
| Publication type | Full report |
| Study design | Cluster randomized trial, unit of allocation: practice |
| Participants | Patients: 840 received allocated intervention, 50.4% female  Clinicians: 8 PHCs, cluster size for each PHC (primary health care center.): 107, 112, 98, 96, 149, 70, 105, 108 respectively, no demographics  Setting: outpatient clinics in Telangana, India |
| Interventions | DSS was a software that helped the physician to (1) undertake a thorough evaluation of risk factors that hypertensive patients may have for developing a CVD; (2) classify the risk level based on data entered by the physician… (3) follow a software-prompted algorithmic guideline-based drug management (which was developed based on Indian hypertension guidelines II [2007])18; and (4) give alerts on the counseling on lifestyle changes and adherence to medication. |
| Outcomes | Primary outcome: mean change in systolic blood pressure (SBP) from baseline to 12 months was the primary endpoint  Duration: 12 months |
| Uptake outcome | CDSS used for 747 of 781 eligible patients (95.6%) |
| Notes |  |

| Andruchow JE, et al. A randomized controlled trial of electronic clinical decision support to reduce unnecessary CT imaging for patients with suspected pulmonary embolism. Canadian Journal of Emergency Medicine. 2018 May;20(S1):S32-3. | |
| --- | --- |
| Publication type | Conference Abstract |
| Study design | Cluster randomized controlled trial, unit of allocation: physicians |
| Participants | Patients: 9609 patients, no demographics  Practitioners: 94 MDs, no demographics  Setting: 4 urban adult EDs and 1 urgent care center |
| Interventions | CDS enabled calculation of patient-specific information including Wells score, PERC score, age-adjusted D-dimer, and prediction for pre-test risk of PE and imaging recommendations |
| Outcomes | Primary outcome: CTPA utilization  Duration: 8 months (when uptake reported) |
| Uptake outcome | From authors: Intervention physicians triggered CDS 1,829 times for eligible suspected PE patients, and voluntarily interacted with it 823 times (45.0%) |
| Notes |  |

| Arts DL, et al. Effectiveness and usage of a decision support system to improve stroke prevention in general practice: a cluster randomized controlled trial. PLoS One. 2017 Feb 28;12(2):e0170974. | |
| --- | --- |
| Publication type | Full report |
| Study design | Cluster randomized trial, unit of allocation: practice |
| Participants | Patients: 522 patients with atrial fibrillation analyzed, mean age 73  Clinicians: 39 general practitioners in 18 clusters were included in the trial, no demographic details provided  Setting: Outpatient general practices, Netherlands |
| Interventions | CDS plugin incorporated into electronic medical record, and automatically activated using event-based triggers. Notifications were shown in a floating window for decision rules, which could be clicked or ignored |
| Outcomes | Primary outcome: Atrial fibrillation treatment guideline adherence  Duration: 11 months (240 active working days) |
| Uptake outcome | 188 of 3848 notifications shown were clicked on by clinicians (4.9%) |
| Notes |  |

| Atlas SJ, et al. A cluster-randomized trial of a primary care informatics-based system for breast cancer screening. Journal of general internal medicine. 2011 Feb;26(2):154-61. | |
| --- | --- |
| Publication type | Full report |
| Study design | Cluster randomized trial, unit of allocation: practice |
| Participants | Patients: 3054 women in intervention practices, mean age 53.7 (SD 7.9)  Clinicians: 64 physicians in intervention arms, mean age 47.4, 48% female  Setting: Outpatient practices |
| Interventions | Web page listing eligible patients overdue for mammogram, with CDS information to help provider decide if patient contact is needed, and electronic checkboxes used to initiate or defer actions. Accessed through hyperlink and EHR |
| Outcomes | Primary outcome: proportion of overdue women undergoing mammography  Duration: 12 months |
| Uptake outcome | 65 of 70 providers used the system (92.9%) |
| Notes |  |

| Atlas, SJ. et al. The medication metronome: A health it system to improve medication management and laboratory monitoring for chronic diseases. In Journal of General Internal Medicine. 2014; Vol. 29, pp. S230-S230 | |
| --- | --- |
| Publication type | Conference abstract |
| Study design | RCT |
| Participants | Patients: 2031 patients in intervention group, no details  Clinicians: 22 physicians in intervention group, no details  Setting: two sites in a primary care network |
| Interventions | Integrated into an existing electronic health record (EHR), the tool enabled primary care physicians (PCPs) to schedule future laboratory monitoring when initiating or adjusting doses of medications for diabetes, hypertension, and/or hyperlipidemia management |
| Outcomes | Primary outcome: the percentage of follow‐up time that a patient was at or below risk factor goal  Duration: 12 months |
| Uptake outcome | Metronome interface was used for 22% of 659 prescriptions (145/659) |
| Notes |  |

| A: Ballard DW, et al. Optimizing clinical decision support in the electronic health record: clinical characteristics associated with the use of a decision tool for disposition of ED patients with pulmonary embolism. Applied clinical informatics. 2016;7(3):883. | |
| --- | --- |
| Publication type | Full report |
| Study design | Pragmatic cluster (non-randomized) trial, unit of allocation: ED site |
| Participants | Patients: 512 encounter patients at active sites  Clinicians: 319 practitioners at active sites, demographics not broken down by site type  Setting: Emergency departments in the Kaiser Permanente Northern California healthcare system |
| Interventions | Active arm CDSS: system to calculate PESI score for patients with pulmonary embolism. Automatically imports information for calculation and launched by user click in EHR |
| Outcomes | Primary outcome: appropriate activation of CDSS  Duration: 8 months |
| Uptake outcome | CDSS used 346/512 times (68%) |
| Notes |  |

| B: Ballard DW, et al. Optimizing clinical decision support in the electronic health record: clinical characteristics associated with the use of a decision tool for disposition of ED patients with pulmonary embolism. Applied clinical informatics. 2016;7(3):883. | |
| --- | --- |
| Publication type | Full report |
| Study design | Pragmatic cluster (non-randomized) trial, unit of allocation: ED site |
| Participants | Patients: 150 encounter patients at passive sites  Clinicians: 70 full time physicians at passive sites, demographics not broken down by site type  Setting: Emergency departments in the Kaiser Permanente Northern California healthcare syster |
| Interventions | Passive arm CDSS: system to calculate PESI score for patients with pulmonary embolism. Automatically imports information for calculation and launched by user click in EHR |
| Outcomes | Primary outcome: appropriate activation of CDSS  Duration: 8 months |
| Uptake outcome | CDSS used 20/150 times (13%) |
| Notes |  |

| A: Blecker S, et al. Interrupting providers with clinical decision support to improve care for heart failure. International journal of medical informatics. 2019 Nov 1;131:103956. | |
| --- | --- |
| Publication type | Full report |
| Study design | Randomized controlled trial |
| Participants | Patients: 465 patients in interruptive alert group, mean age 69.2 (SD 13.9), 30.8% female  Clinicians: no details  Setting: urban academic medical center, inpatient population |
| Interventions | Interruptive pop-up alert triggered to suggest prescribing an ACE inhibitor, integrated into the hospital’s EHR. |
| Outcomes | Primary outcome: discharge utilization of ACE inhibitors or ARBs  Duration: 12 months |
| Uptake outcome | 157/387 (40.6%) interruptive alerts responded to (event) |
| Notes |  |

| B: Blecker S, et al. Interrupting providers with clinical decision support to improve care for heart failure. International journal of medical informatics. 2019 Nov 1;131:103956. | |
| --- | --- |
| Publication type | Full report |
| Study design | Randomized controlled trial |
| Participants | Patients: 493 patients in non-interruptive alert group, mean age 71.9 (SD 14.2), 33.3% female  Clinicians: not discussed  Setting: urban academic medical center, inpatient population |
| Interventions | Non-interruptive alert available to suggest prescribing an ACE inhibitor, integrated into the hospital’s EHR. |
| Outcomes | Primary outcome: discharge utilization of ACE inhibitors or ARBs  Duration: 12 months |
| Uptake outcome | 57/435 (13.1%) non-interruptive alerts responded to |
| Notes |  |

| Bosworth HB, et al. Patient education and provider decision support to control blood pressure in primary care: a cluster randomized trial. American heart journal. 2009 Mar 1;157(3):450-6. | |
| --- | --- |
| Publication type | Full report |
| Study design | Cluster randomized trial, unit of allocation: provider work groups |
| Participants | Patients: 301 patients in CDS and CDS + patient behavioural intervention, mean age 63, 98% male  Clinicians: 34, no more details  Setting: primary care clinic of the Durham VA Medical Center |
| Interventions | Computer assisted medication DSS delivered through the EHR at the point of care detailing BP control and listing personalized treatment recommendations |
| Outcomes | Primary outcome: BP control  Duration: 24 months |
| Uptake outcome | CDSS was interacted with 528 of 929 times it was displayed (57%) during patient visits by providers |
| Notes |  |

| Bourgeois FC, et al. Impact of a computerized template on antibiotic prescribing for acute respiratory infections in children and adolescents. Clinical pediatrics. 2010 Oct;49(10):976-83. | |
| --- | --- |
| Publication type | Full report |
| Study design | Cluster randomized controlled trial, unit of allocation: practice |
| Participants | Patients: 9409 patients in intervention group, mean age 7.6 years, 50.7% female  Clinicians: 112 clinicians including physicians and nurse practitioners  Setting: outpatient, Boston (US) |
| Interventions | CDS system integrated into EMR that provides both a clinical management decision aid and documentation aid for acute respiratory infections |
| Outcomes | Primary outcome: Antimicrobial use during visits for acute respiratory infection  Duration: 6 months |
| Uptake outcome | CDSS was used during 419 visits, accounting for 2.8% of all ARI visits (14964 total) |
| Notes |  |

| Boutis K, et al. Effect of the Low Risk Ankle Rule on the frequency of radiography in children with ankle injuries. CMAJ. 2013 Oct 15;185(15):E731-8. | |
| --- | --- |
| Publication type | Full report |
| Study design | Interrupted time series with pair-matched control design |
| Participants | Patients: 1055 patients at intervention sites, mean age between 11.6 and 12.7 years, 39.9-53.4% male  Clinicians: 28 physicians, no age/sex demographics provided  Setting: Emergency department |
| Interventions | Computerized decision support system for physicians to enter key clinical variables that would automatically generate the recommendation of the ankle rule for radiography |
| Outcomes | Primary outcome: Proportion of eligible patients who received radiography  Duration: 12 months of active intervention |
| Uptake outcome | During intervention phases, the decision support system was used to record data for 89.8% of patients (386 of 430 patients) |
| Notes |  |

| Co JP, et al. Electronic health record decision support and quality of care for children with ADHD. Pediatrics. 2010 Aug 1;126(2):239-46. | |
| --- | --- |
| Publication type | Full report |
| Study design | Cluster randomized controlled trial, unit of allocation: practice |
| Participants | Patients: 206 pediatric patients with ADHD in intervention group, mean age 13.1, 29.1% female  Clinicians: 42 physicians in the intervention group, 43% female, 17.8 mean years since graduating medical school  Setting: outpatient, Eastern Massachusetts (US) |
| Interventions | Decision support reminders and ADHD note templates integrated into EMR, automatically generated at appropriate patient visits |
| Outcomes | Primary outcome: ADHD visits and quality of ADHD care documentation  Duration: 8 months |
| Uptake outcome | CDSS was used by 14 of 42 eligible physicians (33.3%) |
| Notes |  |

| Cox JL, et a. Integrated Management Program Advancing Community Treatment of Atrial Fibrillation (IMPACT-AF): a cluster randomized trial of a computerized clinical decision support tool. American heart journal. 2020 Jun 1;224:35-46. | |
| --- | --- |
| Publication type | Full report |
| Study design | Cluster randomized trial, unit of allocation: practice |
| Participants | Patients: 590 patients in intervention group, mean age 72.5 (SD 10.1), 59.5% male  Clinicians: 104 practitioners in intervention group  Setting: Primary care outpatient practices in Nova Scotia, Canada |
| Interventions | Individualized, auto-generated, evidence-based, and prioritized atrial fibrillation care recommendations (alerts), in a web-based, point-of-care CDS system |
| Outcomes | Primary outcome: Atrial fibrillation related ED visit or unplanned cardiovascular hospitalization  Duration: 12 months |
| Uptake outcome | Total Alerts: n=3409, total Alerts Responded to: n=2081 (61.0%) (from contacting study authors) |
| Notes |  |

| Diaz MC, et al. A Provider-Focused Intervention to Promote Optimal Care of Pediatric Patients With Suspected Elbow Fracture. Pediatric emergency care. 2018 Jan 23. | |
| --- | --- |
| Publication type | Full report |
| Study design | Randomized controlled trial in one health system |
| Participants | Patients: 255 pediatric patients with elbow fracture  Clinicians: 14 emergency department and urgent care physicians  Setting: emergency department, Delaware/Florida (US) |
| Interventions | CDSS included triggered electronic prompts with reminders about examination elements and documentation tools embedded in the EMR |
| Outcomes | Primary outcome: Documentation of neurovascular and musculoskeletal (NV/MSK) assessment  Duration: 3.5 months |
| Uptake outcome | 14 of 14 (100%) of intervention physicians used the CDSS in the care of the 25 patients studied |
| Notes |  |

| Eckman MH, et al. Impact of an atrial fibrillation decision support tool on thromboprophylaxis for atrial fibrillation. American heart journal. 2016 Jun 1;176:17-27. | |
| --- | --- |
| Publication type | Full report |
| Study design | Cluster randomized trial, unit of allocation: practice |
| Participants | Patients: 801 patients with atrial fibrillation, mean age 70.2, 44% female  Clinicians: 35 clinicians from 6 practices  Setting: outpatient, Cincinnati (US) |
| Interventions | Web site accessed through email link providing patient-specific recommendations for AF management and risk stratification |
| Outcomes | Primary outcome: Proportion of patients with antithrombotic therapy discordant with recommendations  Duration: 11 months |
| Uptake outcome | CDSS used in 240 of 801 patients in the intervention group (25.5%) |
| Notes |  |

| Forrest CB, et al. Improving adherence to otitis media guidelines with clinical decision support and physician feedback. Pediatrics. 2013 Apr 1;131(4):e1071-81. | |
| --- | --- |
| Publication type | Full report |
| Study design | Cluster randomized controlled trial, unit of allocation: practice |
| Participants | Patients: children with otitis media; female 47%, 55779 study visits total  Clinicians: 122 pediatricians from 16 practices, female 70%  Setting: Outpatient practices, US (Philadelphia, New Jersey, Delaware) |
| Interventions | Web service integrated into EMR visit navigator (Epic), including data entry tools, cognitive aids, and workflow aids facilitating order entry. |
| Outcomes | Primary outcome: Comprehensive care of otitis media (adherence to guidelines, pain treatment, adequate diagnostic evaluation, and prescription of amoxicillin as a first line therapy)  Duration: 21 months |
| Uptake outcome | Table 2 in Fiks et al: CDS use 6752 visits, no CDS use 34,639 visits (total visits 41,391) |
| Notes | Uptake data from: Fiks AG, et al. Adoption of electronic medical record‐based decision support for otitis media in children. Health services research. 2015 Apr;50(2):489-513. |

| Goergen SK, et al. Can an evidence‐based guideline reduce unnecessary imaging of road trauma patients with cervical spine injury in the emergency department?. Australasian radiology. 2006 Dec;50(6):563-9. | |
| --- | --- |
| Publication type | Full text |
| Study design | Prospective non-randomized controlled trial with historical controls |
| Participants | Patients: 353 study group patients, median age 32 (IQR 23-45), 45.1% female  Clinicians: no details  Setting: emergency department |
| Interventions | The imaging guideline also converted to computerized decision-  support software (DSS), which was linked to a trial database. The ED physicians were asked to use the DSS to assist their management of all  patients with cervical spine trauma, both as an aide memoire but also so that their responses to the guideline questions could be recorded in the trial database. |
| Outcomes | Primary outcome: cervical spine imaging in patients with acute blunt  cervical spine trauma  Duration: 12 months |
| Uptake outcome | Of the 353 patients of the study, 141 (40%) were managed with the assistance of the DSS. The questions of the imaging guideline were answered only by ED physicians who used DSS (patient) |
| Notes |  |

| Gonzales R, et al. A cluster randomized trial of decision support strategies for reducing antibiotic use in acute bronchitis. JAMA internal medicine. 2013 Feb 25;173(4):267-73. | |
| --- | --- |
| Publication type | Full report |
| Study design | Cluster randomized controlled trial, unit of allocation: practice |
| Participants | Patients: 3991 visits with adolescents and adults with uncomplicated acute bronchitis  Clinicians: 41 providers from 11 practices, 90.2% MDs or DOs  Setting: outpatient, Pennsylvania (US) |
| Interventions | CDSS featured a structured template for documentation, prognostication tools, and electronic order sets based on inputted information, automatically generated when triage nurses entered the relevant information into the EMR |
| Outcomes | Primary outcome: Antibiotic use rates for uncomplicated bronchitis  Duration: 6 months |
| Uptake outcome | Smartset was opened 819 times for 11827 electronic alerts (6.9%) (event) |
| Notes |  |

| Gupta S, et al. The Electronic Asthma Management System (eAMS) improves primary care asthma management. European Respiratory Journal. 2019 Apr 1;53(4). | |
| --- | --- |
| Publication type | Full report |
| Study design | Interrupted time-series study |
| Participants | Patients: 890 patients seen in intervention period, mean age 47.3 (SD 17.2), 71.0% female  Clinicians: 18 physicians and 1 nurse practitioner; 65.2% female  Setting: Outpatient general practice clinics, Ontario Canada |
| Interventions | EMR integrated CDSS based on patient specific asthma questionnaire, providing asthma action plan creation, and advice about asthma control assessment and medication escalation/de-escalation |
| Outcomes | Primary outcome: asthma action plan delivery  Duration: 12 months |
| Uptake outcome | Clinicians accessed the CDSS in 174 of the 505 patients (34.4%) in whom actions were required |
| Notes |  |

| A: Hendrix KS, et al. Pediatricians' responses to printed clinical reminders: does highlighting prompts improve responsiveness?. Academic pediatrics. 2015 Mar 1;15(2):158-64. | |
| --- | --- |
| Publication type | Full report |
| Study design | Cluster randomized controlled trial, unit of allocation: clinic |
| Participants | Patients: total number of patients not indicated  Clinicians: total number of practitioners not indicated  Setting: outpatient pediatric clinics in Indianapolis, USA |
| Interventions | Child Health Improvement through Computer Automation (CHICA) system, which uses scanned patient questionnaires to create patient-specific guideline-based physician paper prompts at the point-of-care (highlighted prompts in this intervention arm) |
| Outcomes | Primary outcome: Response to highlighted prompts vs. non-highlighted prompts  Duration: 3 months |
| Uptake outcome | Physicians responded to highlighted prompts 672/1076 times (62%) |
| Notes |  |

| B: Hendrix KS, et al. Pediatricians' responses to printed clinical reminders: does highlighting prompts improve responsiveness?. Academic pediatrics. 2015 Mar 1;15(2):158-64. | |
| --- | --- |
| Publication type | Full report |
| Study design | Cluster randomized controlled trial, unit of allocation: clinic |
| Participants | Patients: total number of patients not indicated  Clinicians: total number of practitioners not indicated  Setting: outpatient pediatric clinics in Indianapolis, USA |
| Interventions | Child Health Improvement through Computer Automation (CHICA) system, which uses scanned patient questionnaires to create patient-specific guideline-based physician paper prompts at the point-of-care (non-highlighted prompts in this intervention arm) |
| Outcomes | Primary outcome: Response to highlighted prompts vs. non-highlighted prompts  Duration: 3 months |
| Uptake outcome | Physicians “responded” to unhighlighted prompts 712/1161 times (61%) |
| Notes |  |

| Hetlevik I, et al. Implementing clinical guidelines in the treatment of diabetes mellitus in general practice: Evaluation of effort, process, and patient outcome related to implementation of a computer-based decision support system. International journal of technology assessment in health care. 2000 Jan;16(1):210-27. (Hetlevik_1) | |
| --- | --- |
| Publication type | Full report |
| Study design | Cluster randomized trial, unit of allocation: center |
| Participants | Patients: 499 patients with diabetes mellitus in intervention group  Clinicians: 24 GPs (8 female, 16 male) in intervention group  Setting: outpatient general practice clinics in Norway |
| Interventions | External computer program accessible from main EHR, guiding doctors in diagnosis, history taking, physical examination, additional tests, and treatment for DM, HTN and hypercholesterolemia |
| Outcomes | Primary outcome: use of CDSS, HbA1c level, serum cholesterol, and blood pressure  Duration: 18 months |
| Uptake outcome | CDSS used partially or completely in 52 of 380 patients (14%) by intervention GPs |
| Notes |  |

| Hetlevik I, et al. Implementing clinical guidelines in the treatment of hypertension in general practice: evaluation of patient outcome related to implementation of a computer-based clinical decision support system. Scandinavian journal of primary health care. 1999 Jan 1;17(1):35-40. (Hetlevik_2) | |
| --- | --- |
| Publication type | Full report |
| Study design | Cluster randomized trial, unit of allocation: health center |
| Participants | Patients: 984 patients with hypertension in the intervention group, mean age 64.5 years  Clinicians: 24 GPs in 17 health centers in the intervention group  Setting: outpatient clinics |
| Interventions | CDSS was an external computer program containing modules that guided clinicians in diagnostics, history and additional test taking, physical examination, and treatments for hypertension, diabetes, and hypercholesterolemia |
| Outcomes | Primary outcome: blood pressure  Duration: 18 months |
| Uptake outcome | CDSS used in 104 of 867 patients (12.0%) |
| Notes |  |

| Kahan NR, Waitman DA, Berkovitch M, Superstine SY, Glazer J, Weizman A, Shiloh R. Large-scale, community-based trial of a personalized drug-related problem rectification system. American Journal of Pharmacy. 2017 Mar 1;9(2):41-6. | |
| --- | --- |
| Publication type | Full report |
| Study design | Non-randomized controlled trial |
| Participants | Patients: 18920 patients in intervention group, mean age 76.4 (SD 7.4), 60% female  Clinicians: 369 physicians in intervention group, mean age 52.7 (SD 9.8), 32% female  Setting: outpatient practices in Israel |
| Interventions | Drug-Drug Interaction Plus (DDI+) decision support system. The DDI+ output is based on a unique technology that synchronizes and integrates pharmacodynamic, pharmacokinetic, and pharmacogenetic data from leading international databases along with patient-specific factors accessed from the patient’s EHR in real time. It is a web-based system that interfaces and operates in the background of an EHR |
| Outcomes | Primary outcome: physician access rates, alert resolution rates, and resource utilization of their patients  Duration: 6 months |
| Uptake outcome | At least 1 episode of a “red alert” was documented during 104,711 visits. Physician voluntary access rates during these visits was observed to be 12.9% (n = 13,534).  During the 6-month study period, 272/369 primary physicians (78.6%) accessed the system at least once. |
| Notes |  |

| Kharbanda EO, et al. Evaluation of an electronic clinical decision support tool for incident elevated BP in adolescents. Academic pediatrics. 2018 Jan 1;18(1):43-50. | |
| --- | --- |
| Publication type | Full report |
| Study design | Cluster randomized trial, unit of allocation: practice |
| Participants | Patients: 607 teenaged patients, mean age 13.7, 51.7% female  Clinicians: no details  Setting: large Midwestern medical group outpatient clinics |
| Interventions | TeenBP, web-based EHR-linked CDS designed assign patients automatically into actionable categories based on their current and prior BPs, BMI, past medical history and recent lab testing. Provides alerts/best practice advisories in the EHR based on this data, to both nurses and medical providers |
| Outcomes | Primary outcome: return for follow-up BP within 30 days  Duration: 12 months |
| Uptake outcome | For 290 patients that the CDS link was displayed, provider opened the CDS for 67 (23%) overall |
| Notes |  |

| Kuilboer MM, et al. Computed critiquing integrated into daily clinical practice affects physicians’ behavior. Methods of information in medicine. 2006;45(04):447-54. | |
| --- | --- |
| Publication type | Full report |
| Study design | Cluster randomized controlled trial, unit of allocation: practice |
| Participants | Patients: mean enrolled patients in intervention practices 4865, mean age 38.4, 50.4% male  Clinicians: Sixteen practices, involving 20 general practitioners, were assigned to the intervention group, mean age 46.5  Setting: general practices in Delft region |
| Interventions | The decision-support system AsthmaCritic provides the general practitioner with patient-specific feedback based on data from the electronic patient record. AsthmaCritic is a non-inquisitive critiquing system that solely relies on the data it receives from the electronic patient record; that is, AsthmaCritic does not ask for additional data entry. As soon as the physician has recorded all data pertaining to a patient visit, AsthmaCritic evaluates whether the patient has asthma or COPD, reviews the physician’s treatment of asthma and COPD, and generates feedback. That is, the physician makes his or her decisions, and AsthmaCritic subsequently critiques these decisions. |
| Outcomes | Primary outcome: average number of contacts, FEV1 (forced expiratory volume), and peak-flow measurements per asthma/COPD patient per practice; and, the average number of antihistamine, cromoglycate, deptropine, and oral bronchodilator prescriptions per asthma/COPD patient per practice  Duration: 5 months of intervention |
| Uptake outcome | MDs read critiques 769 of 10532 eligible visits (7.3%) |
| Notes |  |

| Lee NJ, et al. The effect of a mobile clinical decision support system on the diagnosis of obesity and overweight in acute and primary care encounters. Advances in Nursing Science. 2009 Jul 1;32(3):211-21. | |
| --- | --- |
| Publication type | Full Text |
| Study design | RCT |
| Participants | Patients: 807 patient encounters in experimental group; 58.5% female, mean age 47.8 (SD 17.9)  Clinicians: 13 nurses in experimental group, no further details  Setting: New York-Presbyterian Hospital and multiple ambulatory care sites in the Greater New York City area |
| Interventions | Personal digital assistant-based log with obesity decision support features. Decision support is designed to enhance obesity screening (reminder), offer automatic calculation of the BMI, and to help document obesity-related risk factors, as well as provide guideline information through context-specific links (infobuttons) |
| Outcomes | Primary outcome: obesity related diagnoses and false negative rates  Duration: 8 months |
| Uptake outcome | Decision support system was used 79 times to generate an obesity-related diagnosis, not used 12 times when a diagnosis was still generated, and not used 51 times when a diagnosis should have been generated but wasn’t. Therefore, uptake over total eligible uses is 79/142 (55.6%) |
| Notes |  |

| Lester WT, et al. Randomized controlled trial of an informatics-based intervention to increase statin prescription for secondary prevention of coronary disease. Journal of general internal medicine. 2006 Jan;21(1):22-9. | |
| --- | --- |
| Publication Type | Full report |
| Study design | RCT |
| Participants | Patients: 118 patients in intervention group, mean age 64.3(SD 14.5), 48% female  Clinicians: 14 physicians, 36% female  Setting: academic outpatient practices in Boston |
| Interventions | Cholesterol FastTrack uses automated population surveillance to trigger an e-mail dynamically linked to the EHR, serving as a standalone interactive document providing clinical context and decision support with order entry capability independent of a clinical visit |
| Outcomes | Primary outcome: changes in hyperlipidemia prescriptions  Duration: 12 months |
| Uptake outcome | 117 of 118 (99%) FastTrack emails sent were opened, read, and completed by participating MDs |
| Notes |  |

| Linder JA, et al. An electronic health record–based intervention to improve tobacco treatment in primary care: a cluster-randomized controlled trial. Archives of internal medicine. 2009 Apr 27;169(8):781-7. (Linder 1) | |
| --- | --- |
| Publication type | Full text |
| Study design | Cluster randomized trial |
| Participants | Patients: 5293 patients in the intervention group, mean age 48 (SD 14), 60% female  Clinicians: 207 clinicians in intervention group, 55% female  Setting: outpatient primary care practices in Massachusetts |
| Interventions | We developed and implemented a 3-part electronic health record enhancement: (1) smoking status icons, (2) tobacco treatment reminders, and (3) a decision support Tobacco Smart Form that facilitated the ordering of medication, documentation, and fax and e-mail counseling referrals. |
| Outcomes | Primary outcome: proportion of documented smokers who made contact with a smoking cessation counselor  Duration: 9 months |
| Uptake outcome | 90 of 207 clinicians used the tobacco smart form once or more (44.0%) |
| Notes |  |

| Linder JA, et al. Documentation-based clinical decision support to improve antibiotic prescribing for acute respiratory infections in primary care: a cluster randomised controlled trial. Journal of Innovation in Health Informatics. 2009;17(4):231-40. (Linder 2) | |
| --- | --- |
| Publication type | Full Text |
| Study design | Cluster RCT |
| Participants | Patients: 64321 patients in the intervention group, mean age 49 (SD 17), 61% female  Clinicians: 262 clinicians in intervention group, mean age 39 (SD 12), 55% female  Setting: outpatient primary care clinics in Massachusetts |
| Interventions | EHR-integrated, documentation based clinical decision support system for the care of patients with ARIs, the ARI Smart Form. The ARI Smart Form includes six components: entry of clinical information; patient data display; diagnosis selection; presentation of treatment options with integrated decision support; printing of patient handouts and access to supporting medical literature. |
| Outcomes | Primary outcome: antibiotic prescribing rate for ARIs  Duration: 7 months |
| Uptake outcome | The ARI smart form was used in 6% of ARI visits (742/11954) (visit)  In intervention clinics, 33% (86/262) of clinicians used the ARI smart form at least once |
| Notes |  |

| Mazzaglia G, et al. Effects of a computerized decision support system in improving pharmacological management in high-risk cardiovascular patients: A cluster-randomized open-label controlled trial. Health Inform J. 2016;22(2):232-47. | |
| --- | --- |
| Publication type | Full text |
| Study design | Cluster randomized trial, unit of allocation: practice |
| Participants | Patients: 14,238 patients in the intervention group, 46.9% female, mean age 70.4 (SD 11.2)  Clinicians: 106 GPs in intervention group  Setting: outpatient general practice in Italy |
| Interventions | Alerting computerized decision support system integrated into standard EHR reminding users to initiate pharmacologic management for patients with high CV risk and information on drug-drug interactions |
| Outcomes | Primary outcome: proportion of patients prescribed with cardiovascular drugs and days of drug–drug interaction exposure  Duration: 12 months |
| Uptake outcome | Among 106 GPs in the intervention group, 65 (61.4%) truly maintained the CDSS during follow-up |
| Notes |  |

| McDonald MV, et al. Outcomes of clinical decision support (CDS) and correlates of CDS use for home care patients with high medication regimen complexity: a randomized trial. J Eval Clin Pract. 2016;22(1):10-9 | |
| --- | --- |
| Publication type | Full text |
| Study design | Cluster randomized trial, unit of allocation: nurse |
| Participants | Patients: 2225 patients in intervention, 61% female, mean age 68.4 (SD 14.1)  Clinicians: 165 nurses in intervention, 83% female, mean age 44.7 (SD 9.8)  Setting: outpatient home care, US |
| Interventions | The CDS consisted of a computerized algorithm that identified high MRC patients, electronic clinician alerts, and a care management module. The CDS intervention consisted of three computer-automated components: (i) an algorithm that identified patients with high MRC and thus at increased potential for a serious medication problem or adverse outcome; (ii) a clinical alert – an email delivered to the nurse’s tablet identifying a specific patient with high MRC and directing the nurse to the “medication regimen complexity care management module”; (iii) a high MRC care management module integrated into the Visit unit of the PCRS with specific recommendations for nursing goals and interventions appropriate to patients with multiple co-morbidities and high MRC. |
| Outcomes | Primary outcome: hospitalization, emergency department (ED) use, and change in high medication regimen complexity  Duration: 2 months follow-up per patient |
| Uptake outcome | Only 42% of the 2,550 patients had an intervention nurse action documented in their record  82% of the 165 intervention nurses documented an action with the MRC care management modules with at least one of their targeted patients |
| Notes |  |

| McGinn TG, et al. Efficacy of an evidence-based clinical decision support in primary care practices: a randomized clinical trial. JAMA Intern Med. 2013;173(17):1584-91 | |
| --- | --- |
| Publication type | Full text |
| Study design | RCT |
| Participants | Patients: 586 patients in intervention group, median age 43(IQR 28), 23.9% female  Clinicians: 168 providers in total  Setting: outpatient primary care practices in New York City |
| Interventions | Clinical prediction rule CDS integrated into the EHR that automatically provided decision support based on individual clinical encounter criteria |
| Outcomes | Primary outcome: changes in provider patterns of ordering antibiotics  Duration: 12 months |
| Uptake outcome | Tool opened a total of 62.8% of 586 triggered opportunities (368/586) |
| Notes | Also used: Mann D, Knaus M, McCullagh L, Sofianou A, Rosen L, McGinn T, Kannry J. Measures of user experience in a streptococcal pharyngitis and pneumonia clinical decision support tools. Applied clinical informatics. 2014;5(3):824. Anaylsis of subet of RCT data for functionality and usability of CDSS; and Li AC, Kannry JL, Kushniruk A, Chrimes D, McGinn TG, Edonyabo D, Mann DM. Integrating usability testing and think-aloud protocol analysis with “near-live” clinical simulations in evaluating clinical decision support. International journal of medical informatics. 2012 Nov 1;81(11):761-72. Usability testing |

| McKie PM, et al. Computerized Advisory decision support for cardiovascular diseases in primary care: a cluster randomized trial. The American journal of medicine. 2020 Jun 1;133(6):750-6. | |
| --- | --- |
| Publication type | Full text |
| Study design | Cluster randomized trial, unit of allocation: care team |
| Participants | Patients: Study patient sample: n=16,310 patients, mean age 54 (20); 62% female  ­­­­Clinicians: 55 clinicians in intervention group  Setting: outpatient primary care practices in Minnesota |
| Interventions | CDSS determined if they had heart failure with reduced ejection fraction, hyperlipidemia, or atrial fibrillation; and if so, was the patient receiving guideline-recommended treatment. In the intervention group, an alert was visible in the medical record if there was a discrepancy between current and guideline recommended treatment. Clicking the alert displayed the treatment discrepancy and recommended treatment |
| Outcomes | Primary outcome: the percentage of discrepancies between previsit treatment and guideline-recommended treatment that were resolved within 7 days of the patient visit  Duration: 6 months |
| Uptake outcome | Usage of the CDSS: only 19% of alerts were reviewed. From figure 1 there were 92 alerts for HF, 649 for hyperlipidemia, and 54 for Afib, so denominator is 795 |
| Notes | Also used: Kessler ME, Carter RE, Cook DA, Kor DJ, McKie PM, Pencille LJ, Scheitel MR, Chaudhry R. Impact of electronic clinical decision support on adherence to guideline-recommended treatment for hyperlipidaemia, atrial fibrillation and heart failure: protocol for a cluster randomised trial. BMJ open. 2017 Dec 1;7(12):e019087. |

| McLaughlin D, et al. Office-based interventions for recognizing abnormal pediatric blood pressures. Clinical pediatrics. 2010 Apr;49(4):355-62. | |
| --- | --- |
| Publication type | Full report |
| Study design | Cluster randomized trial, unit of allocation: clinic |
| Participants | Patients: 1223 records reviewed, 571 in the CDS group (no specific demographics for this denominator provided)  Practitioners: more than 40 pediatricians, no details  Setting: outpatient pediatric clinics in Ohio |
| Interventions | PDA application that calculated BP percentile for values entered by nursing staff, displaying a flag for significant values, and automatically printed a report attached to the medical record |
| Outcomes | Primary outcome: physician recognition of elevated BP  Duration: not reported |
| Uptake outcome | 31% (176/571) records contained the PDA receipt |
| Notes |  |

| Meigs JB, et al. A controlled trial of web-based diabetes disease management: the MGH diabetes primary care improvement project. Diabetes Care. 2003 Mar 1;26(3):750-7. | |
| --- | --- |
| Publication type | Full text |
| Study design | RCT |
| Participants | Patients: 307 patients in intervention group, 55.1% female, mean age 68 (SD 12)  Clinicians: 12 providers in intervention group, 33% female  Setting: outpatient internal medicine clinic in Boston |
| Interventions | Web-based Disease Management Application (DMA) displays interactive patient-specific clinical data, treatment advice, and links to other web-based care resources. |
| Outcomes | Primary outcome: glycemic control, cholesterol control, BP control  Duration: 12 months |
| Uptake outcome | The DMA was used for 42% of scheduled patient visits. Total patients in intervention group 307, so 129/307 |
| Notes |  |

| O'Connor PJ, et al. Impact of electronic health record clinical decision support on diabetes care: a randomized trial. Ann Fam Med. 2011;9(1):12-21. | |
| --- | --- |
| Publication type | Full text |
| Study design | RCT |
| Participants | Patients: 1194 patients in intervention group, mean age 57.0 (SD 10.7), 46.7% female  Clinicians: 20 primary care physicians in intervention group, mean age 49.2 (SD 9.9)  Setting: outpatient general medicine clinics in Minnesota |
| Interventions | Diabetes Wizard implementation included the following changes in clinic workflow at intervention clinics: (1) The rooming nurse enters blood pressure readings into EHR as usual. (2) If the patient has diabetes, the rooming nurse opens the Diabetes Wizard in the EHR with a single click on the navigation bar, prints the EHR-generated Diabetes Wizard form (Figure 1), and closes the form in the EHR. (3) The rooming nurse places the printed form on top of the visit summary sheet on the examination room door. (4) The physician reviews the available diabetes treatment options printed on the form just before entering the  room and proceeds with the visit. (5) After the visit but before closing the encounter, the physician opens the Diabetes Wizard form in the EHR visit navigator and completes the brief visit resolution form. |
| Outcomes | Primary outcome: preintervention to postintervention change in hemoglobin A1c, blood pressure, and LDL cholesterol levels  Duration: 6 months |
| Uptake outcome | The intervention group used the EHR-based decision support system at 62.6% of all office visits made by adult patients with diabetes. Looking at Fig 3, we estimate that the total number of uses was ~2800 in the 6 months of the full intervention study, so total visits can be estimated to be 4473 based on this percentage |
| Notes |  |

| Paulsen MM, et al. Effects of using the MyFood decision support system on hospitalized patients' nutritional status and treatment: A randomized controlled trial. Clinical Nutrition. 2020 Dec 1;39(12):3607-17. | |
| --- | --- |
| Publication type | Full text |
| Study design | RCT |
| Participants | Patients: 49 patients in intervention group 29% female, mean age 50 (SD 15)  Clinicians: no details about participating nurses  Setting: inpatient hospital in Norway |
| Interventions | MyFood is comprised of an app for patients and a website for nurses and includes functions for dietary recording, evaluation of intake compared to requirements, and a report to nurses including tailored recommendations for nutritional treatment and a nutritional care plan for documentation |
| Outcomes | Primary outcome: change in body weight during hospitalization  Duration: 9 months |
| Uptake outcome | 60/120 nurses had some time logged into the MyFood website |
| Notes | Also used development report: Paulsen MM, Hagen ML, Frøyen MH, Foss-Pedersen RJ, Bergsager D, Tangvik RJ, Andersen LF. A dietary assessment app for hospitalized patients at nutritional risk: development and evaluation of the MyFood app. JMIR mHealth and uHealth. 2018;6(9):e175. |

| Reed H, et al. Impact of a Best Practice Alert Linking Clostridium difficile Infection Test Results to a Severity-Based Treatment Order Set. In: Open Forum Infectious Diseases 2018 Nov (Vol. 5, No. Suppl 1, p. S79). | |
| --- | --- |
| Publication type | Conference abstract (poster presentation) |
| Study design | Controlled before and after study |
| Participants | Patients: 65 cases, mean age 58.5 (SD 14.1)  Clinicians: no details provided  Setting: inpatient |
| Interventions | Best practice alert (BPA) in the electronic medical record linking a positive test result to guideline-based CDI orders for those not on CDI therapy |
| Outcomes | Primary outcome: antibiotic prescribing and provider adherence to practice guidelines  Duration: 12 months |
| Uptake outcome | The BPA was opened in 54% (28/57) of triggered encounters |
| Notes |  |
| A: Reynolds EL, et al. Randomized controlled trial of a clinical decision support system for painful polyneuropathy. Muscle & nerve. 2020 May;61(5):640-4. | |
| Publication type | Full report |
| Study design | RCT |
| Participants | Patients: 1026 patients in BPA + smartset group, mean age 58.3 (SD 15.2), 68% male  Clinicians: 37 neurology providers in BPA + smartset group  Setting: inpatient, US |
| Interventions | Best practice alert (BPA) linked to a Smartset integrated in the electronic medical record, which gave information involving guideline-recommended medications, typical medication pricing, advice to avoid opioid medication use, and link to the AAN guidelines |
| Outcomes | Primary outcome: proportion of patients with uncontrolled nerve pain that were prescribed a guideline-recommended medication  Duration: 12 months |
| Uptake outcome | BPA was not acknowledged in 789/1026 visits in BPA + smartset group, meaning uptake 237/1026 (23.1%) visits |
| Notes |  |
| B: Reynolds EL, et al. Randomized controlled trial of a clinical decision support system for painful polyneuropathy. Muscle & nerve. 2020 May;61(5):640-4. | |
| Publication type | Full report |
| Study design | RCT |
| Participants | Patients: 1671 patients in BPA alone group  Clinicians: 38 neurology providers in BPA alone group |
| Interventions | Best practice alert (BPA) linked to the electronic medical record indicating that nerve pain status should be documented |
| Outcomes | Primary outcome: proportion of patients with uncontrolled nerve pain that were prescribed a guideline-recommended medication  Duration: 12 months |
| Uptake outcome | BPA was not acknowledged in 1139/1671 visits in BPA alone group, so uptake 536/1671 (32%) |
| Notes |  |
| Rindal DB, et al. Computer-assisted guidance for dental office tobacco-cessation counseling: a randomized controlled trial. Am J Prev Med. 2013;44(3):260-4 | |
| Publication type | Full report |
| Study design | Cluster randomized trial, unit of allocation: clinic |
| Participants | Patients: 263 patients in intervention group, 58.3% female, mean age 45.9 (SD 15.3)  Clinicians: 7 clinics  Setting: outpatient dental clinics |
| Interventions | EHR-based computer assisted tool that suggested scripts for patient discussion around smoking cessation. Scripts tailored to patients by a rule-based algorithm based on patient-specific variables |
| Outcomes | Primary outcome: patient reports of whether providers: (1) asked them  if they smoked; (2) asked about interest in quitting; (3) talked about reasons for quitting; (4) talked about strategies for quitting; or (5) referred them for additional quit-smoking assistance via a toll-free, statewide, telephone quitline  Duration: 5 months |
| Uptake outcome | Scripts were used by hygienists and dentists in intervention clinics at 195/263 (74%) visits |
| Notes |  |
| Robbins GK, et al. Efficacy of a clinical decision-support system in an HIV practice: a randomized trial. Annals of Internal Medicine. 2012;157(11):757-66. | |
| Publication type | Full report |
| Study design | Cluster randomized trial, unit of allocation: provider |
| Participants | Patients: 506 patients in intervention arm  Clinicians: 33 providers had patients randomized  Setting: One HIV clinic at Massachusetts General Hospital |
| Interventions | Virology FastTrack, a CDSS that generates alerts to notify HIV outpatient providers of adverse events. “Interactive alerts” were designed to notify providers of new adverse events or missed appointments via each provider’s EMR “home page,” patient-specific EMR page. Interactive alerts provided key clinical information and a mechanism to request follow-up appointments and/or laboratory tests |
| Outcomes | Primary outcome: CD4 positive lymphocyte (CD4) count increase  Duration: 12 months |
| Uptake outcome | 90% of 1152 interactive alerts were acknowledged by providers (no uptake provided for non-interactive alerts) |
| Notes | The article refers to an appendix, but this is not available online: https://www.ncbi.nlm.nih.gov/pmc/articles/PMC3829692/ |
| A: Rosenbloom, ST, et al. 2005 Effect of CPOE user interface design on user-initiated access to educational and patient information during clinical care. Journal of the American Medical Informatics Association. 2005 Jul 1;12(4):458-73. | |
| Publication type | Full report |
| Study design | RCT |
| Participants | Patients: 4550 patients total, no breakdown  Clinicians: 124 clinicians in intervention group  Setting: inpatient units at Vanderbilt University Medical Center |
| Interventions | PC-POETS CPOE interface that highlighted in bright colour hyperlinks the study-related decision support features in the EHR |
| Outcomes | Primary outcome: rates of opportunities to access and utilization of study-related decision support materials  Duration: 12 months |
| Uptake outcome | There were 278 decision support responses over 240,504 decision support opportunities in the intervention group (0.12%) |
| Notes |  |
| B: Rosenbloom, ST, et al. 2005 Effect of CPOE user interface design on user-initiated access to educational and patient information during clinical care. Journal of the American Medical Informatics Association. 2005 Jul 1;12(4):458-73. | |
| Publication type | Full report |
| Study design | RCT |
| Participants | Patients: 4550 patients total, no breakdown  Clinicians: 78 clinicians in control group  Setting: inpatient units at Vanderbilt University Medical Center |
| Interventions | PC-POETS CPOE interface that could be accessed through the “bells and whistles” tab of the EHR |
| Outcomes | Primary outcome: rates of opportunities to access and utilization of study-related decision support materials  Duration: 12 months |
| Uptake outcome | There were 18 decision support responses over 178,235 decision support opportunities in the intervention group (0.01%) |
| Notes |  |
| Samore MH, et al. Clinical decision support and appropriateness of antimicrobial prescribing: a randomized trial. Jama. 2005 Nov 9;294(18):2305-14. | |
| Publication type | Full report |
| Study design | Cluster randomized trial, unit of allocation: communities |
| Participants | Patients: 6 communities in intervention group, population 32,490, 51% women  Clinicians: 176 clinicians in intervention group  Setting: primary care clinics in rural communities in Utah and Idaho |
| Interventions | Three decision support tools were developed. Two versions were paper-based and 1 was programmed on a PDA. PDA-based CDSS generated diagnostic and therapeutic recommendations on the basis of patient-specific  information that was input about the suspected diagnosis, such as the presence or absence of specific symptoms and signs. Therapeutic recommendations included over-the-counter medications for symptom control as well as  prescription antimicrobials. |
| Outcomes | Primary outcome: community-wide antimicrobial usage  Duration: 9 months |
| Uptake outcome | 125/176 (71%) clinicians used the CDSS tools |
| Notes |  |
| Schnipper JL, et al. Effects of documentation-based decision support on chronic disease management. Am J Manag Care. 2010;16(12 Suppl HIT):SP72-81. | |
| Publication type | Full report |
| Study design | RCT |
| Participants | Patients: 3431 patients in intervention group, mean age 64.5 (SD 13.9), 54% female  Clinicians: 239 primary care practitioners today  Setting: adult primary care practices at Brigham and Women’s Hospital and Massachusetts General Hospital |
| Interventions | The Smart Form is a documentation tool, and as such, has many features in common with other latest-generation EHRs, including the ability to add, edit, and delete coded and/or structured clinical information such as medical problems, medications, and vital signs, and to easily import that information into a visit note. Like some systems that use disease-specific templates, the Smart Form organizes clinical data around certain diseases to facilitate  decision making and also highlights and “requests” missing coded information such as blood pressure, height, weight, and smoking status. The Smart Form also is a CDSS and as such generates output that integrates patient demographic and clinical data with rule-based logic derived from guidelines for the management of CAD and DM. |
| Outcomes | Primary outcome: proportion of deficiencies in care that were addressed within 30 days after a patient visit  Duration: 9 months |
| Uptake outcome | The physicians assigned to the intervention arm used the Smart Form for 5.6% of eligible patients (192/3431 patients) |
| Notes | The article refers to an appendix and another pilot study with the same CDSS. Unable to access either of these online (Schnipper JL, McColgan KE, Linder JA, et al. Improving management of chronic diseases with documentation-based clinical decision support: results of a pilot study. AMIA Annu Symp Proc. 2008:1050.) |
| Schwarz EB, et al. Clinical decision support to promote safe prescribing to women of reproductive age: a cluster-randomized trial. Journal of general internal medicine. 2012 Jul 1;27(7):831-8. | |
| Publication type | Full report |
| Study design | RCT (says cluster but intervention was randomized by physician, not practice) |
| Participants | Patients: 7243 patient encounters in multifaceted CDS group, mean age 34 (SD 10)  Clinicians: 24 physicians in multifaceted CDS, 50% female, mean age 43 (SD 9)  Setting: one academic and one community-based outpatient practice in Western Pennsylvania |
| Interventions | The simple CDS stated “Concern has been raised about the use of this medication during pregnancy” when a potentially teratogenic medication was ordered for a 18–50-year-old female with no indication of sterility in her EMR. The multifaceted CDS expanded upon this by providing a structured order set and tailored alert text that incorporated intake data on women’s pregnancy intentions and contraceptive use (see online appendix). Both CDS systems delivered disruptive alerts requiring PCP acknowledgement. |
| Outcomes | Primary outcome: change in documented provision of family planning services – PCP-reported counseling about the risks of medication induced birth defects and contraception  Duration: 9 months for multifaceted CDS arm |
| Uptake outcome | Only have uptake information for multifaceted CDS arm: PCPs receiving multifaceted CDS accessed the linked order set provided only 16% of the time – denominator 1548 times that CDS was activated and order set available |
| Notes | Online, supplementary data is available: https://www.ncbi.nlm.nih.gov/pmc/articles/PMC3378745/ |

| Semler MW, et al. An electronic tool for the evaluation and treatment of sepsis in the ICU: a randomized controlled trial. Critical care medicine. 2015 Aug;43(8):1595. | |
| --- | --- |
| Publication type | Full report |
| Study design | Randomized controlled trial |
| Participants | Patients: 218 patients in intervention group, mean age 55 years, 54.6% male  Clinicians: No details  Setting: medical and surgical ICUs of one hospital |
| Interventions | Electronic tool capable of importing, synthesizing, an displaying sepsis-related data from the medical record, offering individualized evaluations of sepsis severity and response to therapy, informing users about evidence-based guidelines, and facilitating rapid order entry |
| Outcomes | Primary outcome: time to completion of sepsis bundle elements  Duration: 4 months |
| Uptake outcome | Of 218 patients randomized to the intervention arm, the tool was opened by providers in 126 cases (57.8%) |
| Notes |  |

| Sheibani R, et al. The Effect of a Clinical Decision Support System on Improving Adherence to Guideline in the Treatment of Atrial Fibrillation: An Interrupted Time Series Study. ournal of medical systems. 2018 Feb 1;42(2):26. | |
| --- | --- |
| Publication type | Full report |
| Study design | Interrupted Time Series Study |
| Participants | Patients: 207 AF patients in the post-intervention phase  Clinicians: 10 cardiologists, 70% female, mean age 43.8  Setting: outpatient cardiology offices in the city of Mashhad in Iran |
| Interventions | The intervention was a CDSS designed for the anticoagulant management of AF. This CDSS was designed as a user-friendly mobile application for physicians that calculates risk of AF stroke and of bleeding |
| Outcomes | Primary outcome: adherence to the anticoagulation guideline for the treatment of AF  Duration: 6 months |
| Uptake outcome | the CDSS was used for 88% of AF patients - total patients 182 in post-intervention period (207 minus 25 who had missing data) |
| Notes |  |
| Silbernagel G, et al. Electronic Alert System for Improving Stroke Prevention Among Hospitalized Oral-Anticoagulation-Naïve Patients With Atrial Fibrillation: a Randomized Trial. Journal of the American Heart Association. 2016 Jul 22;5(7):e003776. | |
| Publication type | Full report |
| Study design | RCT |
| Participants | Patients: 455 patients, mean age 74.4 (SD 10.9), 34.1% female  Clinicians: no details  Setting: University hospital Bern, inpatients |
| Interventions | The alert system automatically identified hospitalized patients with AF without an active OAC prescription in the electronic order entry system. The alert system was incorporated into the electronic medical chart and order entry system |
| Outcomes | Primary outcome: rate of adequate OAC prescription at hospital discharge  Duration: 12 months |
| Uptake outcome | In 48 (10.5%) patients from the alert group, physicians used the electronic entry system to calculate the CHA2DS2‐VASc score |
| Notes |  |
| Snooks HA, et al. Support and Assessment for Fall Emergency Referrals (SAFER 1): cluster randomised trial of computerised clinical decision support for paramedics. PloS one. 2014 Sep 12;9(9):e106436. | |
| Publication type | Full report |
| Study design | Cluster randomized trial, unit of allocation: paramedic |
| Participants | Patients: 436 patients analyzed in intervention group, median age 83, 65% female  Clinicians: 17 paramedics in the intervention group  Setting: UK ambulance services |
| Interventions | The health technology evaluated in the experimental arm was CCDS on hand-held tablet computers for use by paramedics to decide whether to take patients who had fallen to an Emergency Department or leave them at home with referral to a community-based falls service |
| Outcomes | Primary outcome: proportion of participants left at scene without conveyance to an Emergency Department and proportion referred to falls services Duration: 12 months |
| Uptake outcome | Intervention paramedics used CCDS for 54 (12.4%) of 436 participants |
| Notes | Supplementary info/study protocol available online: ncbi.nlm.nih.gov/pmc/articles/PMC4162545/ |
| Spirk D, et al. Electronic alert system for improving appropriate thromboprophylaxis in hospitalized medical patients: a randomized controlled trial. Journal of thrombosis and haemostasis. 2017 Nov;15(11):2138-46. | |
| Publication type | Full report |
| Study design | RCT |
| Participants | Patients: 804 patients in alert group, median age 67, 46.6% female  Clinicians: not detailed  Setting: inpatient general internal medicine ward University Hospital Bern |
| Interventions | Computer-based e-alert system for identifying consecutive acutely ill medical patients without therapeutic anticoagulation, and including the Geneva Risk Score calculation tool |
| Outcomes | Primary outcome: rate of appropriate thromboprophylaxis for at least 1 day during hospital stay  Duration: 9 months |
| Uptake outcome | Among the 804 patients in the alert group, 348 had no score calculation by the physician in charge, meaning 456 had the tool used, 56.7% uptake after alert |
| Notes |  |
| Stockwell MS, et al. Registry-linked electronic influenza vaccine provider reminders: a cluster-crossover trial. Pediatrics. 2015;135(1):e75-82. | |
| Publication type | Full report |
| Study design | Cluster randomized cross-over design |
| Participants | Patients: 8481 children had visits, median age 6.5 years (2.1-10.2), 50.3% female  Clinicians: no details provided  Setting: 4 community-based pediatric clinics in New York city |
| Interventions | Non-interruptive vaccination reminder integrated into provider EHR, that allowed vaccination ordering using age-specific options or documentation of reason for vaccine non-administration, with action taken recorded in “Plan” section of EHR notes automatically |
| Outcomes | Primary outcome: percentage of non-up-to-date children vaccinated  Duration: 8 months (2 fall-winter seasons) |
| Uptake outcome | Table 3: over total study period, non-up-to-date alerts occurred 5267 + 13717 times (total 18984), and were deferred or ignored a total of 373 + 709 + 518 + 1379 times (total 2979). Therefore uptake was 16005/18984 (84.3%) |
| Notes | Extra data from: Birmingham E, Catallozzi M, Findley SE, Vawdrey DK, Kukafka R, Stockwell MS. FluAlert: a qualitative evaluation of providers' desired characteristics and concerns regarding computerized influenza vaccination alerts. Preventive medicine. 2011 Mar 1;52(3-4):274-7. |
| Tamblyn R, et al. Evaluating the impact of an integrated computer-based decision support with person-centered analytics for the management of asthma in primary care: a randomized controlled trial. J Am Med Inform Assoc. 2015;22(4):773-83. | |
| Publication type | Full report |
| Study design | Cluster randomized trial, unit of allocation: physician |
| Participants | Patients: 2273 patients in intervention group, 67.8% female, 30% between 5 and 45 years old  Clinicians: 40 physicians in intervention group, 57.5% female  Setting: outpatient family practices in Quebec |
| Interventions | The Asthma Decision Support (ADS) system is integrated into clinician EHR, and incorporates a dashboard alert for out-of-control patients, and decision support for evidence-based asthma management |
| Outcomes | Primary outcome: rate of out-of-control asthma episodes  Duration: 33 months |
| Uptake outcome | In 39.5% of visits for out-of-control asthma, the physicians accessed the ADS system (total 2297 out-of-control visits) |
| Notes |  |

| Tang JW, et al. Electronic tools to assist with identification and counseling for overweight patients: a randomized controlled trial. Journal of general internal medicine. 2012 Aug 1;27(8):933-9. | |
| --- | --- |
| Publication type | Full report |
| Study design | RCT |
| Participants | Patients: 958 patients in intervention group, mean age 46.4 (SD 10.8), 48.5% female  Clinicians: 15 physicians in intervention group  Setting: outpatient academic general internal medicine clinic in Chicago |
| Interventions | The EHR tool set included: a physician point-of-care alert for overweight status, a counseling template to help physicians counsel patients on action plans, and an order set to facilitate entry of overweight as a diagnosis and to order relevant patient handouts. |
| Outcomes | Primary outcome: physician documentation of overweight as a problem  Duration: 6 months |
| Uptake outcome | The counseling template and order set were used in response to 10.7% of alerts.  Email correspondence with authors: interim analysis was accessible which included 520 total alerts, meaning 56/520 alerts were responded to. |
| Notes |  |

| Van Wijk MA, et al. Assessment of decision support for blood test ordering in primary care: a randomized trial. Annals of internal medicine. 2001 Feb 20;134(4):274-81. | |
| --- | --- |
| Publication type | Full report |
| Study design | Cluster randomized trial, unit of allocation: practice |
| Participants | Patients: 78,461 patients enrolled in Bloodlink-guideline practices, mean age 37.1, 49.9% female  Clinicians: 31 general practitioners were assigned to use BloodLink-Guideline, mean age 43.2  Setting: general medicine outpatient practices |
| Interventions | BloodLink-Guideline, provides an overview of available guidelines, with indications for blood test ordering, integrated into the patient electronic record, which then automatically sends the ordered tests and instructions to the laboratory |
| Outcomes | Primary outcome: Average number of blood tests ordered per order form per practice  Duration: 12 months |
| Uptake outcome | Of the 12668 orders placed by practices using the guideline-based CDSS, 9091 (71%) were generated using the decision support system |
| Notes | Other arm (Bloodlink-Restricted) did not include any decision support, so only reporting uptake in the Bloodlink-Guideline arm |

| Williams LK, et al. A cluster-randomized trial to provide clinicians inhaled corticosteroid adherence information for their patients with asthma. J Allergy Clin Immunol. 2010;126(2):225-31, 31.e1-4. | |
| --- | --- |
| Publication type | Full report |
| Study design | Cluster randomized trial, unit of allocation: practice |
| Participants | Patients: 1335 patients in intervention arm, mean age 26.8 (SD 17.4), 55.2% female  Clinicians: 88 providers in intervention arm  Setting: outpatient primary care in Michigan |
| Interventions | Patient-specific medication adherence metrics integrated into the electronic prescribing system, with further detailed information which could be accessed by a link |
| Outcomes | Primary outcome: ICS adherence in last 3-months of the study period  Duration: 12 months |
| Uptake outcome | Table 3: General information was always recorded as “viewed” when providers used the e-Prescribing system, so uptake not possible, but for “detailed information”, providers had to click on a link, and this was done in 52/926 visits in which the link was available to be clicked (5.6% uptake |
| Notes | Supplemental figures that show the CDSS are found online: https://www.jacionline.org/article/S0091-6749(10)00580-4/fulltext |

| Wright A, et al. Improving completeness of electronic problem lists through clinical decision support: a randomized, controlled trial. J Am Med Inform Assoc. 2012;19(4):555-61. | |
| --- | --- |
| Publication type | Full report |
| Study design | Cluster randomized trial, unit of allocation: clinic |
| Participants | Patients: 38,025 patients seen in intervention arm, mean age 47.7 (SD 19.6), 68.0% female  Clinicians: 14 clinics  Setting: primary care practices in Boston |
| Interventions | The intervention was a clinical alert directed to the provider that suggested adding a problem to the electronic problem list based on inference rules, integrated into the electronic medical record system |
| Outcomes | Primary outcome: acceptance rate of the alert  Duration: 6 months |
| Uptake outcome | 13439 alerts were ignored, 3604 overridden, and 7011 accepted, thus uptake (alerts not ignored) was 10615/24054 (44.1%) |
| Notes |  |
